# *In Vitro* Polarization of Colonoids to Create an Intestinal Stem Cell Compartment

**DOI:** 10.1371/journal.pone.0153795

**Published:** 2016-04-21

**Authors:** Peter J. Attayek, Asad A. Ahmad, Yuli Wang, Ian Williamson, Christopher E. Sims, Scott T. Magness, Nancy L. Allbritton

**Affiliations:** 1 Department of Biomedical Engineering, University of North Carolina, Chapel Hill, NC 27599 and North Carolina State University, Raleigh, NC, 27695, United States of America; 2 Department of Chemistry, University of North Carolina, Chapel Hill, NC, 27599, United States of America; 3 Department of Medicine, Division of Gastroenterology and Hepatology, University of North Carolina, Chapel Hill, NC, 27599, United States of America; University of Kentucky, UNITED STATES

## Abstract

The polarity of proliferative and differentiated cellular compartments of colonic crypts is believed to be specified by gradients of key mitogens and morphogens. Indirect evidence demonstrates a tight correlation between Wnt- pathway activity and the basal-luminal patterning; however, to date there has been no direct experimental manipulation demonstrating that a chemical gradient of signaling factors can produce similar patterning under controlled conditions. In the current work, colonic organoids (colonoids) derived from cultured, multicellular organoid fragments or single stem cells were exposed in culture to steep linear gradients of two Wnt-signaling ligands, Wnt-3a and R-spondin1. The use of a genetically engineered Sox9-Sox9EGFP:CAGDsRED reporter gene mouse model and EdU-based labeling enabled crypt patterning to be quantified in the developing colonoids. Colonoids derived from multicellular fragments cultured for 5 days under a Wnt-3a or a combined Wnt-3a and R-spondin1 gradient were highly polarized with proliferative cells localizing to the region of the higher morphogen concentration. In a Wnt-3a gradient, Sox9EGFP polarization was 7.3 times greater than that of colonoids cultured in the absence of a gradient; and the extent of EdU polarization was 2.2 times greater than that in the absence of a gradient. Under a Wnt-3a/R-spondin1 gradient, Sox9EGFP polarization was 8.2 times greater than that of colonoids cultured in the absence of a gradient while the extent of EdU polarization was 10 times greater than that in the absence of a gradient. Colonoids derived from single stem cells cultured in Wnt-3a/R-spondin1 gradients were most highly polarized demonstrated by a Sox9EGFP polarization 20 times that of colonoids grown in the absence of a gradient. This data provides direct evidence that a linear gradient of Wnt signaling factors applied to colonic stem cells is sufficient to direct patterning of the colonoid unit in culture.

## Introduction

Gradients of morphogens, differentiation factors and dietary metabolites are believed to participate in producing a polarized cellular architecture in the crypts of the colon and small intestine by regulating cell proliferation and position [1–5]. Within the crypt, the stem cells reside at the crypt base where they undergo self-renewal and produce proliferative transit amplifying (TA) progenitor cells. TA progenitor cells terminally differentiate and migrate up the long axis of the crypt to eventually populate the colonic epithelium with differentiated lineages: absorptive enterocytes, goblet cells, and enteroendocrine cells [6]. After cells reach the luminal surface, they undergo apoptosis but are replaced by a new generation of cells that maintain the functional absorption, secretion and barrier properties of the epithelium. Intestinal and colonic epithelial stem cells drive renewal of the epithelium every 4–7 days making it one of the most actively self-renewing tissue in the body [7]. It is believed that the orderly movement of cells along the crypt axis from the stem cell compartment to the luminal surface is orchestrated by both intrinsic and extrinsic signaling mechanisms involving gradients of mitogens and morphogens [8, 9]. Crypt patterning is thought to be critically dependent upon the spatial organization of these signals with perturbations of key signaling pathways resulting in disrupted cell positioning and disordered epithelial renewal [9–12]. Despite established roles for certain factors in crypt patterning, less is known as to how discreet levels or gradients of a particular factor regulate crypt morphogenesis [13].

Testing the impact of factor gradients such as Wnt-3a and R-spondin1 on crypt patterning is technically challenging. Studies to date have relied on indirect readouts of gradients using gene and protein expression, and genetic engineering of signal transduction pathways [4, 14–18], but have fallen short of directly testing the influence of gradients on specific cell types. Recently developed culture methods permit the culture of primary colonic epithelial organoids (e.g. colonoids) that possess stem cell and differentiated cellular compartments and self-pattern into crypt units. This major advance in the field now provides a physiologically relevant culture model to test important mechanisms that rely on factor gradients [19, 20]. Colonoid culture requires defined growth conditions that mimic the stem cell niche environment, which promotes stem cell self-renewal and also appropriate differentiation. Essentially, cells are suspended in a 3-D extracellular matrix (Matrigel^TM^), which is rich in collagen and laminin similar to the colonic basal *lamina propria* [19, 20]. The culture system is further supplemented with a mixture of factors including Wnt-3a, R-spondin1/2, Epidermal Growth Factor (EGF), Noggin, and Jagged to maintain stem cell multipotency and self-renewal [4, 20]. When placed in these defined culture conditions, isolated crypts or individual stem cells develop into colonoids with multiple crypt-like buds projecting randomly outward from a central lumen [21]. These buds have a vague resemblance to crypt architecture *in vivo*, and the colonoids often display a disorganized pattern of proliferating cells. The absence of properly formed crypts in the colonoids is likely due to the absence of factor gradients thought to be necessary to define appropriate cell-type locations.

Conventional intestinal stem cell culture systems utilize standard tissue culture dishes which lack of spatial variation in concentrations of chemical factors have hindered the ability to test the influence of fundamental, morphogenic cues in crypt homeostasis and cellular organization. Microfluidic culture systems, however, can readily sustain both simple and complex gradients over sustained times [22–24]. Some of these devices also possess the capability to chemically pattern the environment surrounding cells and tissues [23], so that a device with gradient-forming capabilities can recreate a physiologically-relevant microenvironment for testing mechanistic hypotheses. Microfluidic devices incorporating gut-derived tumor cells or primary mouse intestinal cells have been described for a number of assays on epithelial cells; however, none have been utilized to replicate the complex 3-D architecture of the colonic crypt [25–27]. Efforts have been made to produce scaffolds with a 3-D architecture including crypt-like invaginations in an attempt to place tumor cells into the shape of an intestinal epithelial surface [28–30]. For the most part these studies have employed Caco-2 cells, a tumor derived model gut epithelial cell line, as a surrogate for the intestinal epithelium. While simple to maintain in culture, this tumor cell line has little resemblance to normal epithelium in terms of growth factor response, gene expression and susceptibility to apoptosis making it a poor mimic of the *in vivo* condition [31]. One report used primary murine and human intestinal organoids within a Matrigel layer to promote growth on the surface of a scaffold, but neither the crypt morphology or polarity were recapitulated [30].

Recently our group described a microfluidic gradient device specifically developed for optimizing growth factor concentrations for the efficient culture of colonoids [32]. In this study colonoids were grown within a gradient-channel that exposed each colonoid to a distinct concentration of Wnt and/or R-spondin1 enabling the survey of a wide range of factor concentrations. Colonoids at different ends of the factor gradient displayed distinct phenotypes, *i*.*e*. differentiated or stem/proliferative cells. However, no individual colonoid demonstrated polarized locations of proliferative and differentiated cellular compartments. The absence of cell segregation within a single colonoid was likely due to a shallow gradient imposed across each colonoid effectively placing the entire organoid within the same chemical environment. In the present work, we optimize a microengineered, gradient-forming device to create a steep Wnt-3a and/or R-spondin1 gradient across the length of a single colonoid exposing the cells within the same tissue subunit to different concentrations. The goal was to determine whether a simple, linear gradient of 1 or 2 factors was sufficient to produce polarization of proliferative and differentiated cellular compartments along a colonoid length.

## Materials and Methods

### Transgenic Mouse Models and Isolation of Colonic Crypts

Crypts were isolated from either Sox9EGFP mice or Sox9EGFP-CAGDsRed mice (6–9 weeks old) using previously described methods [32]. The CAGDsRed mouse line ubiquitously expresses the red fluorescent protein DsRed under the control of a chicken beta-actin promoter (CAG). The Sox9EGFP mouse possessed the *Sox9* promoter controlling Sox9EGFP (enhanced green fluorescent protein) expression on a modified bacterial artificial chromosome [33–35]. Mice genetically engineered with this construct express Sox9EGFP in intestinal stem cells and TA cells [33, 35]. Crypts were obtained from colons harvested from mice that were bred, handled and sacrificed under protocols approved by the University of North Carolina at Chapel Hill Institutional Animal Care and Use Committee (http://research.unc.edu/offices/iacuc/). The University of North Carolina at Chapel Hill Institutional Animal Care and Use Committee approved the animal work described in this paper (approval #13–200). Prior to euthanasia, all mice are anesthetized with isoflurane followed by cervical dislocation to minimize any stress or pain. Single intestinal stem cells were obtained from crypts harvested from heterozygous Sox9EGFP:CAGDsRED mice between 6 and 10 weeks of age by fluorescence-activated cell sorting (FACS) (S1 Methods) [34].

### Colonoid Culture

Colonoid culture media (CCM) was prepared as previously described [32] and consisted of a mixture of advanced DMEM/F12 medium (Invitrogen), Wnt-3a (120 ng/mL) and R-spondin1 (175 ng/mL) unless otherwise specified. CCM also contained Noggin (100 ng/mL), EGF (50 ng/mL), Y27632 ROCK inhibitor (10 μM), NAC (1 mM), GlutaMAX (1×), HEPES (10 mM), penicillin (100 unit/mL), and streptomycin (100 μg/mL). Wnt-3A and R-spondin1 were prepared from conditioned medium as described previously or purchased purified from a supplier (R&D Systems, Minneapolis, MN). The CCM was prepared in a bulk volume of 500 mL, split into 6-mL aliquots, and stored at -80°C until use. For crypt culture, 100% Matrigel (Dow Corning, Midland, MI) was used. Crypts were isolated from the distal colon of a mouse as previously described [32]. The crypts were pelleted by centrifugation at 300×G for 90 s. The supernatant was aspirated and the crypts were mixed with cold liquid Matrigel (100% in CCM, 4°C). A 1 mL suspension of freshly isolated crypts (5000 crypts/mL) was added to standard 12-well plates at 4°C. The Matrigel was then polymerized for 15 min at 37°C. After polymerization, 1.5 mL of CCM was overlaid onto the Matrigel.

After 5 days in culture, colonoids were retrieved from the Matrigel using collagenase digestion (15 min, 37°C) and then fragmented using trypsin/EDTA (2 min, 37°C in 0.02 mM trypsin and 0.48 mM EDTA). The slurry was pipetted vigorously for 30 s to break the colonoids into multicellular fragments. These colonoid fragments were then rinsed, counted and re-embedded in Matrigel for culture in CCM. This process was repeated to grow colonoids in continuous culture as well as to prepare fragmented colonoids to load into the microdevice.

### Diffusion-Based Gradient Generation and Characterization

Devices were fabricated as described in S1 Methods. Gradient formation through the Matrigel layer on the device was characterized by imaging the movement of a 40 kDa fluorescein-labelled dextran (Sigma-Aldrich, St. Louis, MO) in 100% Matrigel by time-lapse imaging using an Olympus MVX10 Macroview microscope. Fluorescence images were acquired every 15 min over 24 h to measure gradient formation. The volume of the source and sink was 500 μL and that of the channel was 1.5 μL. Gradient formation over time was modeled using Fick’s Law [36]:

$$C\left(x,t\right)=A+\;\frac{1}{2}C_{O}\;erfc\;\left(\frac{x}{2\surd (Dt)}\right)$$

where A is a constant, *x* ranges from 0 to 1 mm corresponding to the positions along the length of the channel, *t* is time, *D* is the diffusion coefficient, *erfc* is the complementary error function, and *C*_*O*_ is the concentration of the species of interest loaded into the source. COMSOL Multiphysics with finite-element analysis (FEA) was used to model the data. For experiments applying gradients to colonoids, the media in both the source and sink were replaced every 24 h.

### Culture of Colonic Cells in the Microchannel of the Gradient Device

Before use, the device was sterilized with 70% ethanol and rinsed with phosphate buffered saline (PBS) ×5. The gradient-generating region of the device was pre-coated by incubation with 2% Matrigel in PBS for 6 h at 4°C and then rinsed with PBS ×3 prior to loading cells. This pre-coating step resulted in deposition of a thin Matrigel surface layer on the channel walls which improved subsequent cell/Matrigel loading into the gradient region and enhanced adhesion of the gelled plug to the device walls. Cells were mixed with cold liquid Matrigel (100% in CCM, 4°C) and loaded into the device’s gradient-generating region. The Matrigel plug was gelled by incubation at 37°C for 15 min. Once the Matrigel solidified, CCM (500 μl) was immediately added to each reservoir. For experiments in which a gradient was formed, Wnt-3a and/or R-spondin1 were omitted from the CCM added to the sink as indicated in the text.

### Microscopy

Colonoid formation and growth was monitored over time using a Nikon Eclipse TE2000-U microscope fitted with a Photometrics CoolSNAP HQ2 digital camera. Objective lenses used were 10× and 20× with numerical apertures of 0.30 and 0.55, respectively. Fluorescein-dextran diffusion in the microchannel was tracked by wide-field imaging of the entire device using an Olympus MVX10 Macroview fluorescence microscope with a 1.0×, 0.25 N.A. objective and 0.63× demagnification. Confocal images of gradient formation were obtained using a Zeiss CLSM 710 Spectral Laser Scanning Microscope equipped with a 488 nm laser to excite fluorescein.

### Immunofluorescence and EdU Assays

Crypts isolated from a Sox9EGFP-only mouse were used for immunofluorescence staining to avoid interference from the DsRed fluorescence. For immunofluorescence staining, crypts or colonoids were fixed with 4% paraformaldehyde for 20 min, followed by permeabilization with 0.5% Triton X-100 (Thermo-Fisher, Waltham, MA) for 20 min. After rinsing ×3 with PBS containing 100 mM glycine, the sample was incubated in immunofluorescence wash (0.2% Triton X-100, 0.1% BSA, 0.05% Tween-20, 7.7 mM NaN_3_ in PBS and 5% normal goat serum) for 90 min to block nonspecific binding. The polyclonal rabbit α-Muc2 primary antibody (1:200, Life Sciences) in immunofluorescence wash was then incubated with the sample for 12 h at 4°C. Secondary antibody (α-rabbit-Cy3, 1:500, Life Sciences) in immunofluorescence wash was then incubated with the sample for 45 min. All nuclei were stained with Hoechst 33342 (10 μg/mL in PBS) using a 30 min incubation. Microdevices were imaged by brightfield and fluorescence microscopy. An EdU-based assay was also used to identify cells undergoing active DNA synthesis in S-phase of the cell cycle per manufacturer’s protocol (Life Technologies, product #10640).

### Colonoid Segmentation using DsRed or Hoechst 33342

A custom script written in MATLAB (MathWorks; Natick, MA) was used to segment the colonoids by identifying DsRed or Hoechst 33342 positive pixels. Fluorescence images were filtered using a top hat filter with a disk-shaped structuring element to remove background fluorescence and uneven background illumination [37–40]. The images were then thresholded using minimum cross entropy thresholding [38, 39]. In the resultant binary image, all interior holes within objects were filled and objects with a total area less than 1000 μm^2^ were removed to generate a mask of the segmented colonoids (S1 Fig). Brightfield images were then used to remove large cellular debris by applying a Chan-Vese active contour to the brightfield image using the previously generated mask as an initialization [40]. Cellular debris was defined as objects that possessed brightfield segmentation boundaries that were 20% larger than the segmentation boundary obtained from the DsRed or Hoechst fluorescence suggesting an object consisting of noncellular or degrading cellular material. The area of each colonoid was determined and used as a proxy for the total number of cells within each colonoid.

### Measurement of the Percentage of Colonoids Positive for a Fluorophore

Colonoids were identified and segmented as described above based on either DsRed or Hoescht 33342 fluorescence. The fluorescence images of the Sox9EGFP, Muc-2 immunofluorescence staining, or the EdU fluorophore were filtered to reduce background noise (top-hat filtering) and the fluorescence intensity of each pixel previously identified as being within the boundaries of a colonoid (using the DsRed or Hoescht 33342 mask) was summed. The number of pixels positive for EGFP, Muc-2 immunofluorescence staining, or the EdU fluorophore was then quantified based on empirically set fluorescence intensity thresholds for these fluorophores (in comparison to control samples). Based on empiric observation, colonoids were marked as positive for Sox9EGFP, Muc-2 or EdU if 25%, 10%, or 25% of the pixels, respectively, in the colonoid were positive for the fluorophore [32].

### Measurement of Sox9EGFP Polarization in a Colonoid

Colonoids were segmented as described above. The DsRed mask obtained for the segmented colonoids was applied to the Sox9EGFP fluorescence image to identify the colonoid boundaries in the Sox9EGFP image. Sox9EGFP fluorescence intensity was then divided by DsRed intensity for each pixel. This acted to normalize the Sox9EGFP fluorescence with respect to the cell number in different regions of the colonoid. Each colonoid was then cropped from the resultant image. Within each cropped image, the mean intensity of a 20-μm horizontal slice through the center of the colonoid was calculated to generate an intensity profile along the center of the colonoid. The cropped image was rotated by 1 degree about the centroid of the bounding box of the colonoid using nearest neighbor interpolation and the mean intensity of a 20-μm horizontal slice through the center of the colonoid was again calculated. This was repeated for 180 degrees of rotation. A linear fit was performed on each intensity profile to obtain the slope of that profile. The rotated image that produced the largest absolute value of the slope was identified. The angle of the rotated image and sign of the slope determined the direction of colonoid polarization. The absolute value of the slope of the linear fit was used as the magnitude of the polarization (S2 and S3 Figs).

### Measurement of EdU Polarization in a Colonoid

Colonoids were segmented based on Hoechst 33342 and the EdU image divided by the Hoechst 33342 image as described above for Sox9EGFP and DsRed. This acted to normalize the EdU fluorescence with respect to the cell number in different regions of the colonoid. Each colonoid was then cropped from the resultant image. Since a minority of cells stained with EdU, the EdU fluorescence exhibited a punctate distribution in images (unlike Sox9EGFP fluorescence). For this reason the polarization measurements were altered for the EdU-based measurements. For each segmented colonoid, the Hoechst 33342 geographic centroid and the EdU intensity-weighted centroid were identified. The angle of the vector between the two points was used as the angle of polarization. The magnitude of the vector was normalized to the total length of the colonoid (length along the axis of polarization) (S4 Fig). For EdU polarization measurements, the axes were defined as described for the Sox9EGFP polarization measurements.

### Statistics

Boxplots were used to represent the non-normal distribution of colonoid area and Sox9EGFP fluorescence intensity of the developing colonoids [41]. Within the boxplots, stars represented the mean, a bar represented the median, and the upper and lower boxes showed the 75% and 25% percentile of the data, respectively. The whiskers extended to the 5^th^ and 95^th^ percentile with outlying data shown as individual points. The data are presented in the text as medians, first- and third-quartile values for colonoid DsRed area and colonoid Sox9EGFP fluorescence intensity within the regions. For statistical comparison, the data were converted to a normal distribution using a logarithmic transform and then assessed using Q-Q plots for their fit to a normal distribution. The adjusted coefficient of determination (R^2^) values for the Q-Q plots was always ≥ 0.91. Statistical differences between data were identified using a Holm-Sidak t test in the analysis of variance [42]. Data are also presented as average ± standard deviation where appropriate, with the compass plot data being represented as the standard deviation. Propagation of uncertainty using the standard deviation was used to calculate the variation in the Sox9EGFP/EdU polarization angle and magnitude. Once this was found, statistical differences in the compass plot data were assessed using a Holm-Sidek t-test to determine whether the differences in polarization directions of colonoids grown in the presence of specific gradients was statistically significant [43]. Similarly, a Holm-Sidek t-test was used to examine the statistical differences between the percentages of colonoids possessing stem/TA cells (Sox9EGFP), goblet cells (Muc2+) and actively proliferating cells (EdU+) in colonoids cultured in the microchannel and the multiwell plate (S5 Fig). For all statistical analyses, a p-value less than 0.05 was considered to be significant.

## Results and Discussion

### Design and Characterization of Gradient-Microdevice

**Fig 1:**
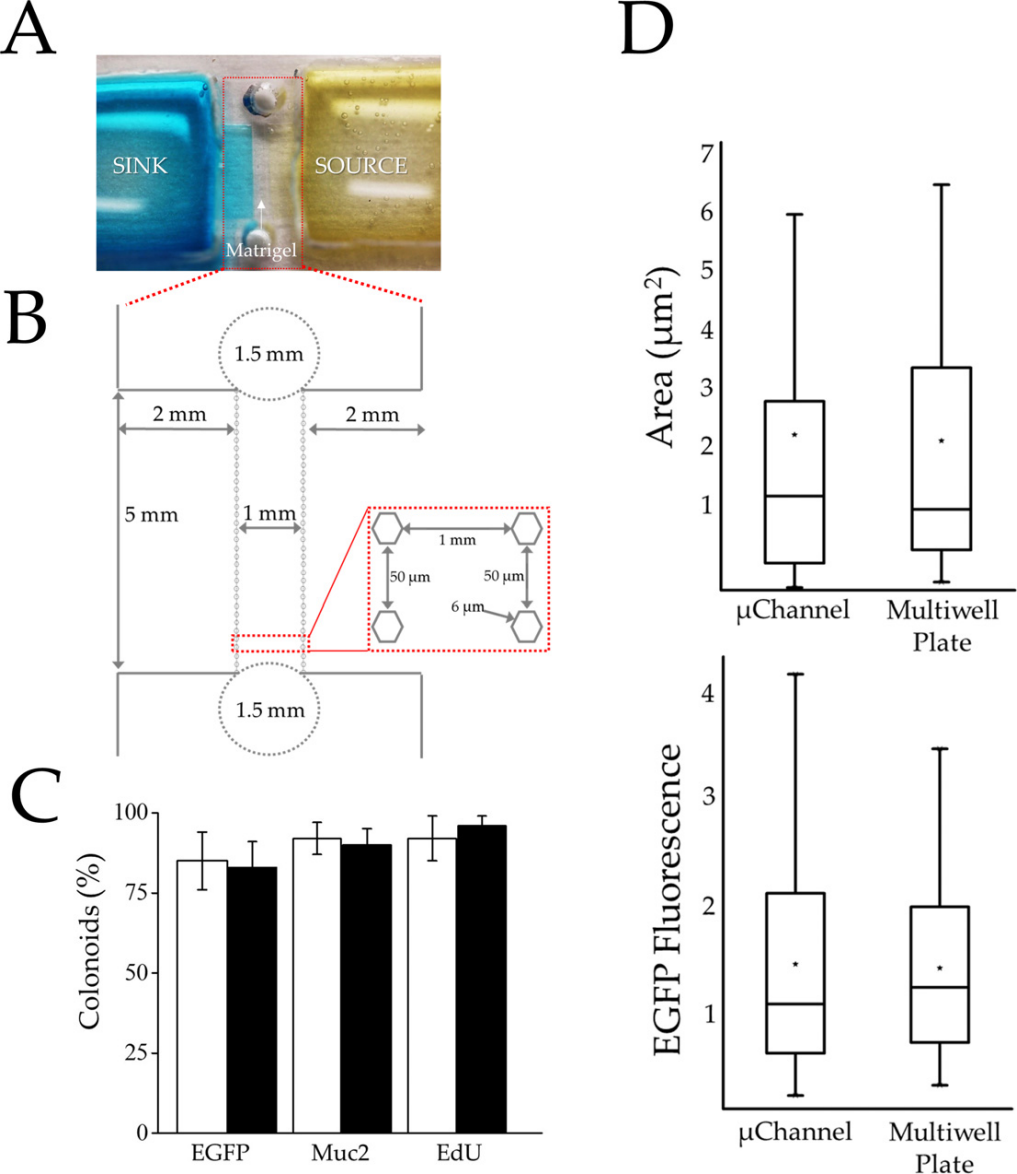
Characterization of the gradient-generating microdevice. (A) Photograph of the device. The Matrigel-filled gradient region resides between the sink (left with blue dye) and source (right with yellow dye) reservoirs. (B) Schematic of the gradient generating microchannel of the device. The 1.5-mm diameter circles mark the ports for loading Matrigel into the central microchannel. (C) Histogram showing percentages of colonoids possessing Sox9EGFP expression (stem/TA cell), exhibiting Muc2 staining (goblet cells) and labeling with EdU (actively proliferating cells) when cultured in the microchannel (black) or conventional multiwell plate (white). (D) Colonoid area (top) and Sox9EGFP fluorescence (bottom) per colonoid are shown after 5 days in culture in either the microchannel or microwell. Boxplots were used to represent the non-normal data distribution. Colonoid area is represented as μm^2^ (× 10^4^) and Sox9EGFP fluorescence intensity is represented as RFUs (× 10^5^). For the boxplots, the black star indicates the mean of the data, the bar shows the median, and the upper and lower boxes represent the 75% and 25% of the data, respectively. The whiskers extend to the 5% and 95% of the data.

Poly(dimethylsiloxane) (PDMS) was selected as the material of choice for the device as PDMS is gas permeable and compatible with colonic stem cell culture [32, 44]. Devices formed from PDMS are also readily fabricated using soft lithography [45]. The device incorporated a central microchannel (1×5×0.3 mm with a volume of 1.5 μL) across which a linear gradient (1 mm in length) was formed between two large fluid reservoirs. The two fluid reservoirs (16×16×5 mm with a volume of 1.3 mL) were placed to either side of the microchannel and served as a source or sink (Fig 1A and 1B and S6 Fig). Matrigel was loaded into the central channel via a small inlet and outlet port (1.5 mm diameter) at the ends of the microchannel. An array of hexagonal posts (250 μm height, 6 μm face and 50 μm inter-post spacing) bounded the sides of the gradient-generating region and acted to localize Matrigel to the central microchannel via surface-tension forces. Every third post was labelled with a number permitting the channel location to be reproducibly identified over time during microscopy.

The diffusion of fluorescein-labeled dextran (40 kDa) was used to characterize the time evolution and stability of a gradient formed across the 1-mm width of the Matrigel-filled microchannel. Fluorescein-dextran was loaded into the source reservoir and the microchannel was imaged over time. By 1 h, a gradient of fluorescence had begun to form across the microchannel decreasing linearly from the concentration in the source to that in the sink. When the temporal evolution of the fluorescence intensity was fit to Fick’s Second Law of Diffusion, a diffusion coefficient of 7.2 ± 0.6 × 10^−11^ m^2^/sec (n = 3 devices) was calculated for the fluorescein-dextran which was similar to that measured by other investigators for the 42-kDa protein vascular epithelial growth factor in Matrigel (7.0 × 10^−11^ m^2^/sec) (S7 and S8 Figs) [46, 47]. To maintain the linear gradient over long time scales (5 days), the source and sink solutions were replaced every 24 h. Modeling the device and solution changes suggested that once a gradient was established, the concentration of a 40 kDa analyte across the microchannel varied by no more than 0.9% over a 5 day period (S9 Fig). The daily reservoir refreshment combined with the 870× volume of the source and sink reservoirs relative to that of the gradient-forming microchannel enabled the source and sink reservoirs to behave as infinite compartments and permit formation of a time-invariant molecular gradient [48]. Similar gradient strategies have been employed successfully by others [48, 49]. These data also suggest that stable, linear gradients of Wnt-3a (39.7 kDa) and R-spondin1 (40.0 kDa) could be formed across the Matrigel-filled microchannel.

### Microchannel devices support colonoid development similar to conventional cultures

Colonoid growth in a standard format (12-well plate) was compared to that on the microdevice in the absence of factor gradients. Dual transgenic reporter mice (Sox9EGFP:CAGDsRed) were used to identify and monitor changes in stem/progenitor cells and differentiated cells. Sox9EGFP marks stem/progenitor cells and CAGDsRed is ubiquitously expressed in all cells. As cell differentiate, DsRed-only expression serves as a proxy marker for differentiated cell types [34]. DsRed+ colonoid area was used to determine colonoid size. Freshly isolated crypts were mixed with Matrigel and loaded into the microchannel. Wnt-3a (60 ng/mL) and R-spondin1 (90 ng/mL) were placed into both the source and sink reservoirs and replenished every 24 h during culture. In parallel, crypts were cultured in a conventional Matrigel patty placed in a multiwell plate and overlaid with media containing 60 ng/mL of Wnt-3a and 90 ng/mL of R-spondin1. The media was replenished every 24 h for both formats.

Of the Sox9EGFP-CAGDsRed crypts plated in the microdevice, 55 ± 14% (avg. ± s.d.) developed into colonoids with a median DsRed area/colonoid of 13,236 μm^2^ (S1 Table). In comparison, 60.0 ± 8.5% of crypts plated and cultured for 5 days in the Matrigel patties developed into colonoids with a median DsRed area/colonoid of 12,752 μm^2^ after 5 days in culture (Fig 1D and S1 Table). The presence of a differentiated cell type producing mucous (Goblet cells) was assayed by immunofluorescence staining of Mucin 2 (Muc-2) after Sox9EGFP crypts were plated in the microdevice and cultured for 5 days [33]. The percentage of colonoids in the microchannel with Goblet cells (Muc-2+ staining in ≥10% of the colonoid area) was 90 ± 5% compared to 92 ± 7% in the multiwell plate (Fig 1C, S5 Fig and S2 Table). These data indicate that microchannels support colonoid development and differentiation similar to convention Matrigel-patty cultures.

To assess the impact of the microchannel devices on proliferative cells, Sox9EGFP crypts were plated into microchannels or the conventional platform and cultured for 5 days. Sox9EGFP expression and EdU were used to assess the numbers of proliferative stem/progenitor cells in all colonoids on the devices. The percentage of colonoids demonstrating Sox9EGFP expression in ≥25% of the colonoid area was similar for the microchannel and conventional multiwell environments with 83 ± 8% and 85 ± 9% of colonoids positive for Sox9EGFP, respectively (S3 Table). The median integrated Sox9EGFP fluorescence/colonoid in the microchannel was 118,822RFUs (S4 Table). In comparison, the median integrated Sox9EGFP fluorescence/colonoid in the multiwell plate was 133,490 RFUs (Fig 1D and S3 Table). A second assay based on an EdU pulse, which marks cells in the S-phase of the cell cycle, was used to quantify proliferating cells [50]. The percentage of colonoids in the microchannel with EdU+ cells (occupying >25% of the colonoid area) was 96 ± 3%, compared to 92 ± 7% in the standard Matrigel patty on the multiwell plate (Fig 1C and S5 Table). These data indicate that microchannels and multiwells yielded colonoids containing similar numbers of proliferating stem/progenitor cells.

### Colonoids do not demonstrate overall polarization in the absence of an external gradient

**Fig 2:**
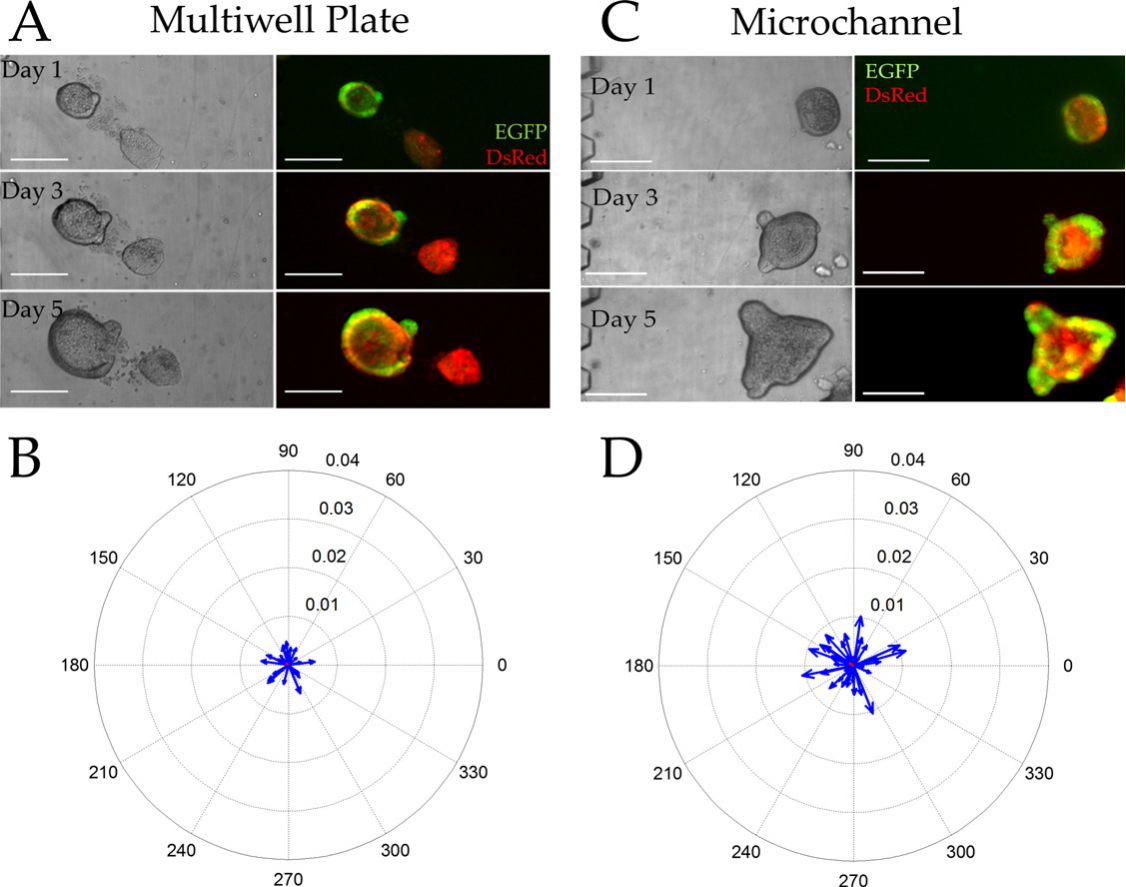
Colonoid properties in the absence of a gradient. (A, C) Brightfield (left) and overlaid red/green fluorescence (right) images of colonoids cultured within a standard multiwell plate (A) or microchannel (C) for 1, 3, and 5 days Scale bars represent 250 μm. (B, D) Compass plots displaying the Sox9EGFP polarization magnitude and angle for individual colonoids cultured in the multiwell plate (B) or microchannel (C) for 5 days (n = 49 colonoids in 10 microchannels and n = 30 colonoids in 5 wells). The blue vectors represent individual colonoids while the average magnitude and angle of the vector is marked in red (poorly visualized due to the near-zero magnitude).

The localization of Sox9EGFP within colonoids in the absence of an external chemical gradient was assessed to determine the extent to which individual colonoids might polarize the stem/progenitor cell compartments on the microchannel devices. Colonoids derived from a Sox9EGFP-CAGDsRed mouse were loaded into microchannels or multiwell plates and cultured in the presence of Wnt-3a (60 ng/mL) and R-spondin1 (90 ng/mL). We developed vector-based computational image analysis pipelines to measure the relative location of Sox9EGFP expressing cells in the colonoid. For the microchannel device, a line perpendicular to the long axis of the microchannel was defined as the line through 0 to 180 degrees. Zero and 180 degrees were arbitrarily defined for each multiwell plate, but was consistent across all wells. A Sox9EGFP polarization vector was calculated by searching for the steepest gradient in Sox9EGFP intensity as the colonoid was rotated through 180 degrees. Using this strategy, an unpolarized colonoid is characterized by a Sox9EGFP vector magnitude that approaches zero while a highly polarized colonoid is near 0.04. For the conventional multiwell platform, the average Sox9EGFP polarization vector possessed a length of 0.0006 ± 0.0006 and an angle of 150 ± 110 degrees. The colonoids in the microchannel displayed an average Sox9EGFP polarization vector with a length of 0.0009 ± 0.0007 and an angle of 152 ± 107 degrees. When the Sox9EGFP polarization angle was examined for each colonoid in both the multiwell plate and microchannel, the angle appeared to be randomly distributed through all quadrants and the magnitude of the vectors near zero. Additionally, the initial location of a colonoid in the microchannel or multiwell plate had no impact on its likelihood of being polarized (S10 Fig and S6 Table). Thus, colonoids cultured under these gradient-free conditions displayed Sox9EGFP fluorescence nearly equally distributed about the colonoid in all locations of both culture formats (Fig 2).

**Fig 3:**
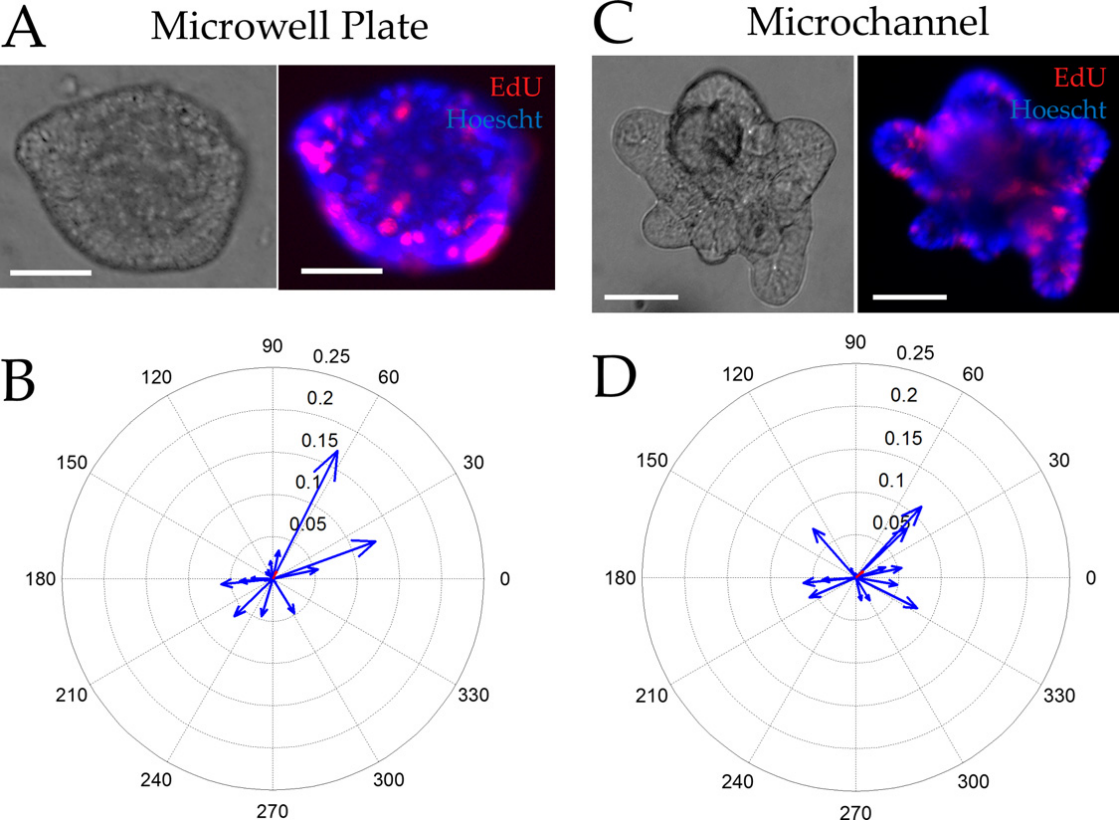
Incorporation of EdU into colonoids after a 2 h pulse in the absence of a gradient. (A,C) Brightfield (left) and overlaid red/blue fluorescence (right) images of colonoids cultured within a multiwell plate (A) or microchannel (C) for 5 days then labeled with EdU (red) and the Hoechst 33342 (blue) (n = 18 colonoids in 5 microchannels and n = 16 colonoids in 3 multiwells). Scale bars represent 50 μm. (B, D) Compass plots displaying the EDU polarization magnitude and angle for individual colonoids (blue) cultured in the multiwell plate (B) or microchannel (C) for 5 days and pulsed with EdU. The average magnitude and angle of the vector can be seen in red (poorly visualized due to the near-zero magnitude).

Using a similar vector-based approach, we assessed the extent to which the proliferative cell compartment (marked by EdU) was polarized in the absence of an external gradient. Colonoids were cultured in both microchannel and conventional platforms for 5 days at which time EdU was added to the culture for 2 h. Since only a small subset of cells were marked by EdU, the algorithm used for Sox9EGFP polarization could not be employed. Instead the geographic centroid and the intensity-weighted centroid were identified and the vector between these two locations was used to assess EdU polarization. Using this algorithm, unpolarized colonoids possessed an EdU vector magnitude approaching zero while the largest possible vector magnitude for highly polarized colonoids was 0.5. For the conventional multiwell plate, the average EdU polarization vector possessed a length of 0.009 ± 0.064 and an angle of 61 ± 26 degrees. The colonoids in the microchannel displayed an average EdU polarization vector with a length of 0.011 ± 0.053 and an angle of 38 ± 41 degrees. As with the Sox9EGFP vectors, the EdU vector magnitudes suggested that in the absence of an external growth factor gradient the rapidly proliferating cells within a colonoid showed no spatial preference (Fig 3).

### A Wnt-3a gradient is sufficient to polarize the stem/progenitor cell compartments

We next sought to test whether formation of a gradient of Wnt-3a, a factor that supports stem cell maintenance, would promote the polarization of the stem/progenitor cell compartment when applied across a single colonoid. To test this, colonoids were loaded into the microchannel and Wnt-3a (75 ng/mL) was added to the source reservoir only. After 5 d of culture under the linear Wnt-3a gradient, the colonoid area, Sox9EGFP expression, and EdU incorporation were measured. The median DsRed area/colonoid in the microchannel device was 14,096 μm^2^ per colonoid (S11 Fig). After 5 days in culture, the colonoid area under the Wnt-3a gradient was not statistically different from that without a gradient in either platform. The median integrated Sox9EGFP fluorescence per colonoid was 73,591 RFUs (S7 Table and S12 Fig). Similar to the DsRed fluorescence, the integrated Sox9EGFP fluorescence per colonoid was not statistically different from that of colonoids in the absence of a gradient. These data suggest that the colonoids under the Wnt-3a gradient possess similar numbers of stem/progenitor cells as the colonoids cultured in the absence of a gradient.

**Fig 4:**
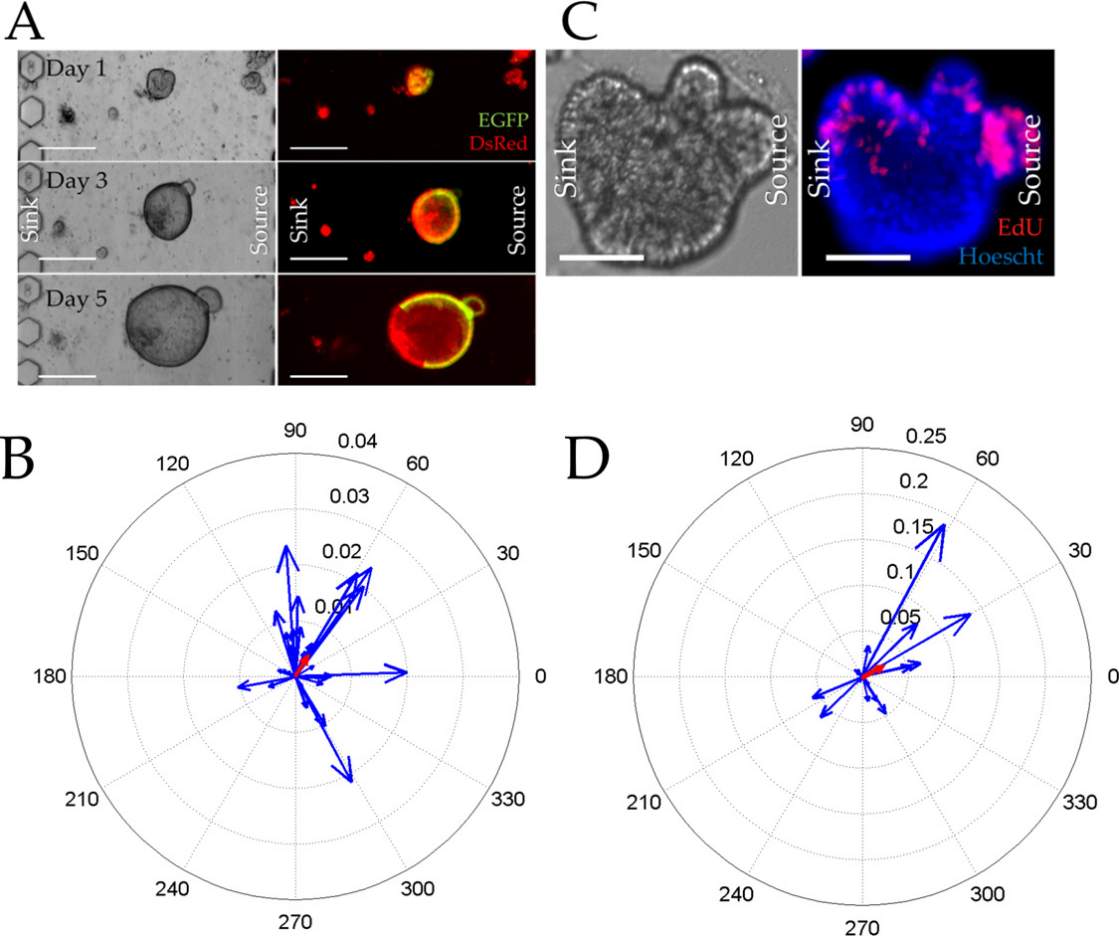
Colonoid growth in the presence of a Wnt-3a gradient across the microchannel. (A) Brightfield (left) and overlaid red/green fluorescence (right) images of colonoids cultured under a Wnt-3a gradient for 1, 3, and 5 d. The scale bar is 250 μm. (B) Compass plot displaying the Sox9EGFP polarization magnitude and angle for individual colonoids cultured under the Wnt-3a gradient for 5 days (n = 28 colonoids on 5 devices). The average magnitude and angle of the vector can be seen in red. (C) Brightfield (left) and overlaid red/blue fluorescence (right) images of colonoids cultured under a Wnt-3a gradient for 5 days then pulse-labeled with EdU (red) for 2 h. Hoechst 33342 fluorescence is shown in blue. The scale bar represents 50 μm. (D) Compass plot displaying the EDU polarization magnitude and angle for individual colonoids (blue) cultured as described in (C) (15 colonoids in 5 microchannels). The average magnitude and angle of the vector can be seen in red.

Although the total size and Sox9EGFP fluorescence per colonoid in the gradient and no-gradient conditions were similar, the distribution of stem or proliferating cells across the colonoid under these two conditions might be distinct. To assess this possibility, the average Sox9EGFP and EdU polarization vectors of the Wnt-3a-gradient-exposed colonoids after 5 d in culture was measured and compared to a no-gradient condition. Colonoids exposed to the Wnt-3a gradient possessed an average Sox9EGFP polarization vector magnitude of 0.0044 ± 0.0019 and an angle of 58 ± 21 degrees, both of which were statistically different from that of colonoids in the microchannel in the absence of a gradient (p<0.05). Of the 28 colonoids surveyed under the Wnt-3a gradient, 22 colonoids (79%) possessed Sox9EGFP polarization vectors facing the Wnt-3a source (Fig 4A and 4B). The larger Sox9EGFP vector magnitude also suggested that the colonoids were more polarized than that without a gradient. However, the average vector was well short of the highly-polarized value of 0.04 so that the colonoids were not under maximal polarization conditions. The presence of Sox9EGFP polarization in a colonoid was not related to its location in the microchannel (S13 Fig and S6 Table). Under the influence of the Wnt-3a gradient, the average EdU polarization vector possessed a length of 0.02 ± 0.06 and an angle of 41 ± 39 degrees. The EdU polarization vector of colonoids exposed to Wnt-3a gradient demonstrated that eleven of the colonoids (73%) polarized the EdU expressing cells toward the Wnt-3a source (Fig 4C and 4D). Thus the majority of the colonoids in the channel responded to the Wnt-3a gradient with their proliferating cells localized to colonoid regions with sufficient Wnt-pathway activation creating discrete stem/TA cell compartment similar to what is seen *in vivo* at the base of the crypt. This data also demonstrates that a Wnt gradient alone is sufficient to polarize a colonoid in the absence of other signaling gradients such as BMP, Noggin, or R-spondin.

### A Wnt-3a/R-spondin1 gradient enhances polarization of the stem/progenitor cell compartment

**Fig 5:**
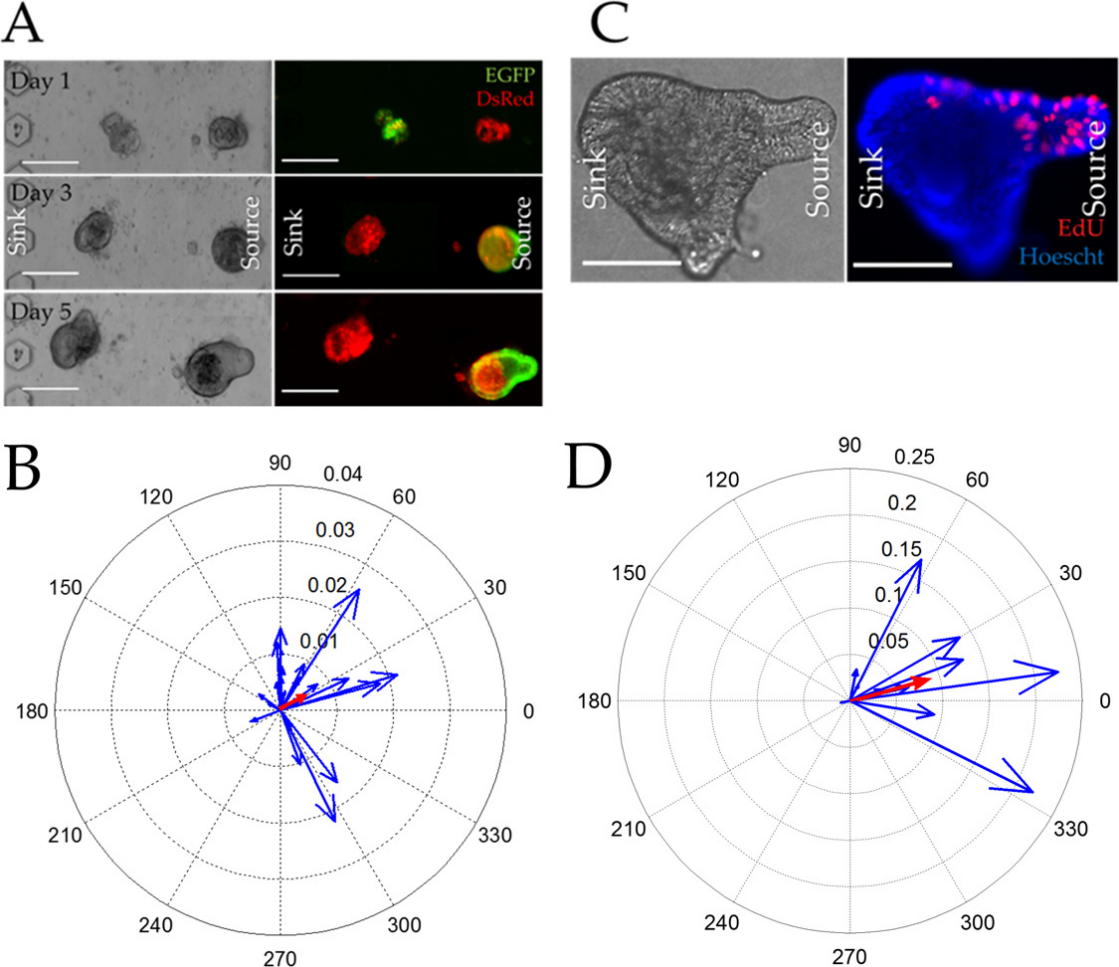
Colonoid growth in the presence of a dual Wnt-3a/R-spondin1 gradient. (A) Brightfield (left) and overlaid red/green fluorescence (right) images of colonoids cultured under a Wnt-3a/R-spondin1 gradient for 1, 3, and 5 days in the microchannel. The scale bar is 250 μm. (B) Compass plot displaying the Sox9EGFP polarization magnitude and angle for individual colonoids (blue) cultured under the Wnt-3a/R-spondin1 gradient for 5 days (n = 33 colonoids from 6 microchannels). The average magnitude and angle of the vector can be seen in red. (C) Brightfield (left) and overlaid red/blue fluorescence (right) images of colonoids cultured the gradient for 5 days then pulse-labeled with EdU (red) for 2 h. Hoechst 33342 fluorescence is shown in blue. The scale bar represents 50 μm. (D) Compass plot displaying the EDU polarization magnitude and angle for individual colonoids (blue) cultured as described in (C) (n = 11 colonoids in 4 microchannels). The average magnitude and angle of the vector can be seen in red.

The highest levels of Wnt activity are thought to exist in the crypt base with the Wnt concentration tapering off in a gradient toward the luminal surface. R-spondin1 (Rspo1) is co-expressed with Wnts in the stem cell zone and functions to potentiate Wnt activity through its receptor LGR5, which is G-protein coupled receptor expressed almost exclusively in colonic stem cells [51]. To determine whether a dual gradient of Wnt-signaling along the microchannel might promote enhanced polarization of proliferative and differentiated cellular compartments, R-spondin1 and Wnt-3a were placed at high concentration in the source reservoir (75 ng/mL Wnt-3a, 110 ng/mL R-spondin1) to generate an environment with a steep factor gradient. After 5 days under a Wnt-3a/R-spondin1 gradient, the median DsRed fluorescence (area/colonoid) was 22,146 μm^2^ and the median Sox9EGFP fluorescence (intensity/colonoid) in the presence of the dual gradient was 105,823 RFUs (S11 and S12 Figs and S8 Table). These values were not statistically different from that in the absence of a gradient or in the presence of a Wnt-3a gradient alone) suggesting that the number of stem/progenitor cells and differentiated cells were not changed in the presence of a Wnt-3a/R-spondin1 gradient. To assess whether a Wnt-3a/R-spondin1 gradient enhanced polarization of the stem/progenitor and differentiated compartments, Sox9EGFP and EdU location was measured in each colonoid under the dual factor gradient. The average Sox9EGFP polarization vector exhibited a magnitude of 0.0049 ± 0.0019 and an angle of 35 ± 31 degrees, which was a statistically significant difference from that of the colonoids in the absence of a gradient, but not significantly different from that of a Wnt-3a gradient alone. Of the 24 colonoids assessed in the dual gradient, 92% possessed a Sox9EGFP vector that orientated toward the source reservoir containing Wnt-3a/R-spondin1 (Fig 5A and 5B). As with the Wnt-3a gradient, the presence of Sox9EGFP polarization in a colonoid was not related to its location in the microchannel (S13 Fig and S6 Table). Analysis of EdU labeling under the Wnt3-a/R-spondin1 gradient revealed an average EdU polarization vector magnitude of 0.09 ± 0.07 and an angle of 15 ± 19 degrees, which was a statistically significant difference from polarization vectors in the absence of a factor gradient or Wnt-3 alone (Fig 5C and 5D). Nearly all colonoids in the Wnt-3a/R-spondin1 gradient exhibited proliferating cells oriented toward the highest Wnt3a/R-spondin1 concentrations similar to what is observed *in vivo* at the base of the crypt.

### Effect of a Wnt-3a and R-spondin1 Gradient on Growth and Polarization of Colonoids Derived from a Single Stem Cell

The experiments above utilized multicellular colonoid fragments as the source material for development of colonoids. While the fragments were small (~30 μm diameter with ~25 cells), the fragments did contain many cell types (differentiated, stem, and TA cells), and thus may have had pre-established cellular interactions that might impact spatial lineage allocation of a colonoid developing under an externally imposed growth-factor gradient. In addition, the colonoid fragments were obtained from continuously cultured colonoids (>1 month). While all evidence to-date indicates that the cells within these colonoids are representative of those *in vivo* and maintain a normal karyotype, it is conceivable that the cultured colonoids differ in an as yet unknown manner from their *in vivo* counterparts [5, 21, 52]. For this reason, single stem cells were isolated from freshly obtained Sox9EGFP-CAGDsRed mouse crypts by fluorescence-activated cell sorting of the stem cells (Sox9EGFPlow:CAG^DsRED^) [34]. The stem cells suspended in Matrigel were loaded into a microchannel and cultured for 5 d in the presence of a Wnt-3a/R-spondin1 gradient. The median DsRed fluorescence area per colonoid was 16,576 μm^2^ so that the area of the 5-day colonoids in the dual gradient was not statistically different from that obtained in the prior experiments using multicellular colonoid fragments (S11 Fig). Similarly, the Sox9EGFP fluorescence/colonoid of the single stem cell-derived colonoids (median of 95,734 at 5 days) did not display a statistically significant difference from that of prior experiments (S12 Fig and S9 Table). These data suggested that colonoids originating from individual stem cells grew robustly, catching up in size and stem/TA cell numbers to that of the cultured colonoid fragments.

**Fig 6:**
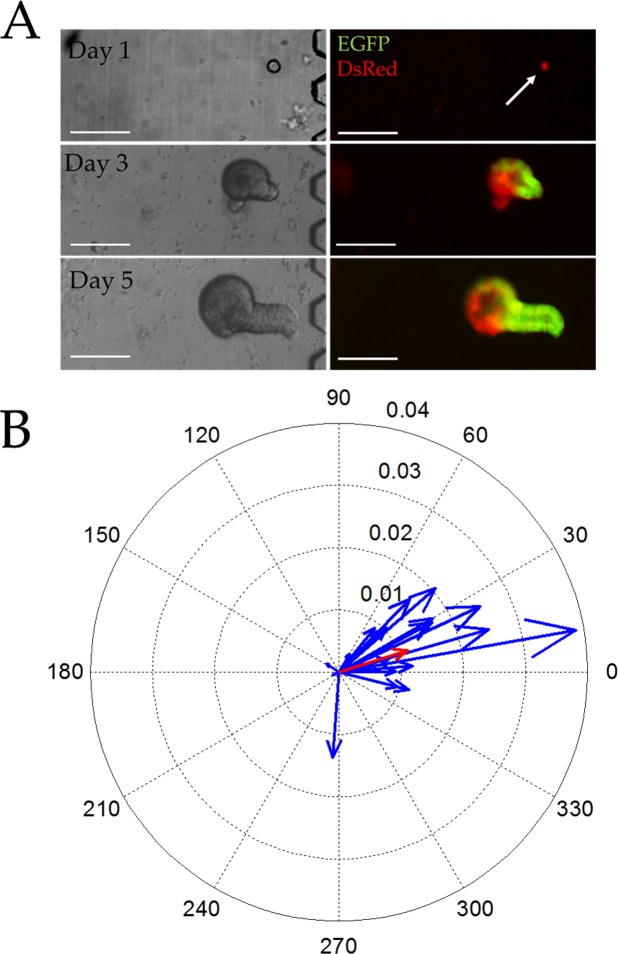
Growth of single stem cells in the presence of a Wnt-3a/R-spondin1 gradient. (A) Brightfield (left) and overlaid red/green fluorescence (right) images of single stem cells cultured under a Wnt-3a/R-spondin1 gradient for 1, 3, and 5 days in the microchannel. The scale bar is 250 μm. (B) Compass plot displaying the Sox9EGFP polarization magnitude and angle for the single colonoids (blue) under the Wnt-3a/R-spondin1 gradient for 5 days (n = 23 colonoids in 5 microchannels). The average magnitude and angle of the vector can be seen in red.

The Sox9EGFP polarization vector for the single-cell-derived colonoids was measured to test the hypothesis that these colonoids might more readily polarize under the Wnt-3a/R-spondin1 gradient since the single cells were initially free of cell-cell interactions. The average Sox9EGFP polarization vector of the single-cell-derived colonoids possessed a magnitude of 0.012 ± 0.002 and an angle of 17 ± 16 degrees which was a statistically significant difference from that of colonoid-fragment-derived colonoids under a Wnt-3a/R-spondin1 gradient (p<0.05). Of the 23 colonoids surveyed in the dual gradient condition, 20 colonoids (87%) possessed Sox9EGFP vectors pointing in the direction of the growth factor source (Fig 6). A similar percentage of colonoids arising from the single cells and colonoid fragments polarized to align with the growth factor gradient; however, colonoids developing from the single stem cells were more highly polarized as evidence by greater vector magnitudes than those arising from the colonoid fragments. In addition, colonoids located near the sink were more likely to be polarized than those located near the source (S14 Fig and S6 Table). The above data suggest that cell interactions within the colonoid fragments exert an additional influence on the behavior of the stem and/or TA cells modifying the colonoid's ability to fully spatially orient in response to environmental cues. The growth-factor gradient concentrations near the sink also appear more appropriate for that required to induce colonoid polarization.

## Conclusions

Basal-luminal polarization of the stem/progenitor and differentiated cellular compartments in the intestinal and colonic crypts is considered to be dictated by gradients of morphogenic factors [1, 4]. A number of studies indirectly demonstrate through gene expression assays that downstream targets of canonical Wnt signaling exist in a graded fashion along the crypt axis [11, 53]. Formal testing of how specific growth factor or chemical gradients influence a number of biological processes like cellular differentiation and crypt patterning have been precluded by limitations in colonic stem cell culture and platforms that enable the generation of steep growth factor gradients. Here, we optimized a platform to introduce tightly controlled steep linear gradients of morphogenic factors, similar to those widely held to be present *in vivo*, across individual colonoids or single stem cells. We demonstrate that imposing a simple linear gradient of Wnt-3a/Rspondin1 is necessary and sufficient to induce polarization of the proliferative and differentiated cellular architecture in colonoid tissue constructs derived from both single colonic stem cells and small multicellular colonoids composed of heterogeneous cell types.

In these studies, a stem and proliferative cell niche was recreated within a single epithelial unit by imposing a simple, linear, 2-factor gradient suggesting that *in vivo*, polarization of Wnt-signaling along the crypt axis is likely to be sufficient to maintain the basal-to-luminal crypt organization. Introduction of a 75 pg/mL/μm gradient of Wnt-3a resulted in polarized colonoids with localization of proliferative stem and TA cells in regions of higher Wnt-3a concentration paralleling the *in vivo* cellular patterning of the intestinal crypt. In this single gradient experiment, R-spondin1, also an activator of the Wnt signaling pathway, was at a uniform concentration of 90 ng/mL throughout the microchannel. It is possible that the R-spondin1 alone provided sufficient Wnt pathway activation to support the Sox9EGFP-expressing cells throughout the colonoids. A combined gradient of Wnt-3a (75 pg/mL/μm) and R-spondin1 (110 pg/mL/μm) acted synergistically to produce enhanced polarization of the colonoid body. The concentration drop over distance for these factor gradients was similar to those reported in the literature for other critical factors controlling a variety of biological processes and organismal development [54–56]. Maintenance of colonoids derived from single stem cells under the combined gradient condition produced the most highly polarized colonoid structure suggesting that the multicellular fragments possessed some internal patterning which limited the ability of colonoids derived from them to maximally respond to morphogenic gradients. These experimental results demonstrate for the first time that a simple linear gradient of growth factors is capable of directing polarization of the cellular architecture along the crypt axis.

## Supporting Information

S1 Fig Example of colonoid segmentation using DsRed fluorescence. (A) Raw images from brightfield microscopy and fluorescence microscopy of EGFP and DsRed of the sample field of colonoids. (B) A threshold for the processed image was automatically determined by minimum cross entropy thresholding.[38] (C) In the resultant binary image, all objects with a total area less than 1000 μm^2^ were removed and all interior holes within objects were filled to generate a mask of the segmented colonoids. (D) Large cellular debris was then removed from the images. Cellular debris was defined objects with brightfield segmentation boundaries that were 20% larger than the segmentation boundary obtained from the red fluorescence channel. Colonoids touching the edges of the image were also removed. (E) Finally, each of the colonoids were labelled with a color code for subsequent measurements on that colonoid. Scale bars are 250 μm.
(JPG)

S2 Fig Example calculation of colonoid EGFP polarization. (A) Images of the DsRed fluorescence, EGFP fluorescence, and EGFP divided by DsRed fluorescence. (B) The EGFP divided by DsRed image was rotated every one degree over 180 degrees. (C) A 20-μm horizontal slice through the center of the colonoid was identified. (D) The intensity profile along the 20- μm slice was calculated and a linear fit was performed on the intensity profile to obtain the slope of the best-fit line.
(JPG)

S3 Fig Identification of the EGFP polarization angle and magnitude. The colonoid rotation angle was plotted against the absolute value of the slope. The rotated image that produced the largest absolute value of the slope was identified. This angle of the rotated image and sign of the slope determined the direction of colonoid polarization. The absolute value of the slope was used as the magnitude of the polarization. In this example the angle of polarization was 138 degrees with a magnitude of 0.01.
(JPG)

S4 Fig Example calculation of colonoid EdU polarization. Shown is the EdU fluorescence image of a colonoid. The geographic centroid (yellow asterisk) was obtained from the Hoechst 33342 image (not shown). The EdU-intensity weighted centroid (red asterisk) was also calculated. The angle of polarization was the angle of the vector (blue arrow) that pointed from the geographic centroid to the intensity weighted centroid. The magnitude of the vector was normalized to the colonoid length (346 μm in this example).
(JPG)

S5 Fig Representative overlaid images of fluorescent stains. EGFP fluorescence is depicted in green in panels A and B. Muc-2 immunofluorescence is shown in red panels C and D while EdU-based fluorescence is also marked as red panels E and F. Hoechst 33342 fluorescence is blue in all panels. Colonoids were cultured for 5 d in a microchannel (A, C, E) or multiwell plate (B, D, F). The scale bars are 150 μm.
(JPG)

S6 Fig Detailed schematic of gradient-forming microdevice. Panel A shows the device of Fig 1A with a dashed black line depicting the location of the cross section shown in panel B.
(JPG)

S7 Fig Fluorescent images of the Matrigel-filled microchannel during gradient formation. Fluorescein-dextran (40 kD) was loaded into the source only. The fluorescence across the microchannel was then measured. A) Microchannel image immediately after fluorescein dextran placement into the microchannel. B-E) Images of the channel at 1 (B), 6 (C), 12 (D), and 24 (E) h after placing fluorescein dextran into the source.
(JPG)

S8 Fig Fluorescence of the Matrigel-filled microchannel over time without reservoir fluid replacement. Fluorescein-dextran (40 kD) was loaded into the source. The fluorescence across the microchannel was immediately measured and is labeled as time 0. The fluorescence was again measured at 1, 6, 12, and 24 h.
(JPG)

S9 Fig Modeling of gradient variation over time with replenishment of the source and sink reservoirs at 24 h. Shown is the fluorescence intensity *vs* the distance along the microchannel at varying time points after replacement of the source and sink reservoir fluids (marked as time 0 on the graph). The gradient was allowed to form for 24 h and then the reservoir fluids replaced at time 0. The boundary of the sink and Matrigel was designated as 0 μm and that at the Matrigel and source as 1000 μm.
(JPG)

S10 Fig Dependence of the EGFP polarization vector on the centroid location of the colonoid fragment in the multiwell plate or microchannel without a gradient. The centroid location of the colonoid fragment in the multiwell plate or microchannel (between the source and sink) was calculated from the DsRed fluorescence. The centroid distance from the Matrigel:sink interface (located at 0 μm) was then plotted against the x-component of the EGFP polarization vector. The Matrigel:source interface was located at 1000 μm. A straight line was fit to the data points (solid line). The images are data from the multiwell plate (A) or microchannel without a gradient (B).
(JPG)

S11 Fig DsRed fluorescence area of colonoids under Wnt-3a alone or Wnt-3a/Rspondin1 gradients. Boxplots were used to represent the DsRed fluorescence area of the colonoids for two gradient conditions (Wnt-3a alone and Wnt-3a/Rspondin1 (W+R)) starting with colonoid fragments and the W+R condition starting with single cells. The non-normal distribution of the colonoid area is represented as μm^2^ (× 10^4^). For the boxplots, the red star indicates the mean of the data, the horizontal line shows the median, and the upper and lower boxes represent the 75% and 25% of the data, respectively. The whiskers extend to the 5% and 95% with the individual points showing outliers. (A) Day 1 and (B) Day 5.
(JPG)

S12 Fig Integrated EGFP intensity of colonoids under Wnt-3a alone or Wnt-3a/Rspondin1 gradients. Boxplots were used to represent the integrated EGFP intensity of the colonoids for the two gradient conditions (Wnt-3a alone and Wnt3a/Rspondin1 (W+R)) starting with colonoid fragments and the W+R condition starting with single cells. The non-normal distribution of the colonoid integrated EGFP fluorescence intensity is represented as RFUs (× 10^5^). For the boxplots, the black star indicates the mean of the data, the bar shows the median, and the upper and lower boxes represent the 75% and 25% of the data, respectively. The whiskers extend to the 5% and 95% with the individual points showing outliers. (A) Day 1 and (B) Day 5.
(JPG)

S13 Fig Dependence of the EGFP polarization vector on the centroid location of colonoids in a Wnt-3a alone or Wnt-3a/Rspondin1 gradient. The centroid location of the colonoid in the microchannel (between the source and sink) was calculated from the DsRed fluorescence. The centroid distance from the Matrigel:sink interface (located at 0 μm) was then plotted against the x-component of the EGFP polarization vector. The Matrigel:source interface was located at 1000 μm. A straight line was fit to the data points (solid line). The images are data from the Wnt-3a (A) or Wnt-3a/Rspondin1 (B) gradient.
(JPG)

S14 Fig Dependence of the EGFP polarization vector on the colonoid microchannel location for colonoids originating from a single stem cell placed in a Wnt-3a/Rspondin1 gradient. The stem-cell location in the microchannel between the source and sink was plotted against the x-component of the EGFP polarization vector. A straight line was fit to the data points (solid line).
(JPG)

S1 Methods Fabrication of the 1-mm Gradient Device; Isolation of Single Colonic Stem Cells; COMSOL Modeling. (DOCX)

S1 Table Area occupied by each colonoid in a 2-D image slice in the absence of a gradient after 1 and 5 days of culture in the microchannel or multiwell plate. Six devices were used for each microchannel experiment and three wells for each multiwell-plate experiment.
(DOCX)

S2 Table Percentage of each colonoid with Muc-2 immunofluorescence in a 2-D image slice in the absence of a gradient after 5 days of culture on the microchannel and multiwell plate. For the microchannel, n = 20 colonoids in 5 microchannels and for the multiwell plate, n = 20 colonoids in 3 wells.
(DOCX)

S3 Table Percentage of each colonoid with EGFP fluorescence in a 2-D image slice in the absence of a gradient after 5 days of culture on the microchannel and multiwell plate. (DOCX)

S4 Table EGFP fluorescence intensity of colonoids in a 2-D image slice in the absence of a gradient after 1 and 5 days of culture in the microchannel or multiwell plate. (DOCX)

S5 Table Percentage of each colonoid with EdU fluorescence in a 2-D image slice in the absence of a gradient after 5 days of culture on the microchannel and multiwell plate. (DOCX)

S6 Table The correlation coefficient, *p*-value, slope and adjusted R^2^ value for plots of the colonoid microchannel location vs the x component of the EGFP polarization vector. (DOCX)

S7 Table Integrated EGFP intensity of a 2-D image slice of colonoids developed within a Wnt-3a gradient after 1 and 5 days of culture on the microdevice. (DOCX)

S8 Table Integrated EGFP intensity of a 2-D image slice of colonoids developed within a Wnt-3a + Rspondin1 gradient after 1 and 5 days of culture on the microdevice. (DOCX)

S9 Table Integrated EGFP intensity of a 2-D image slice of colonoids developed from single cells within a Wnt-3a + Rspondin1 gradient after 1 and 5 days of culture on the microdevice. (DOCX)
