# Supplementary figures and images for: In Vitro Polarization of Colonoids to Create an Intestinal Stem Cell Compartment
Source: PLoS One. 2016 Apr 21;11(4):e0153795. doi: 10.1371/journal.pone.0153795 (PMC4839657; doi:10.1371/journal.pone.0153795)

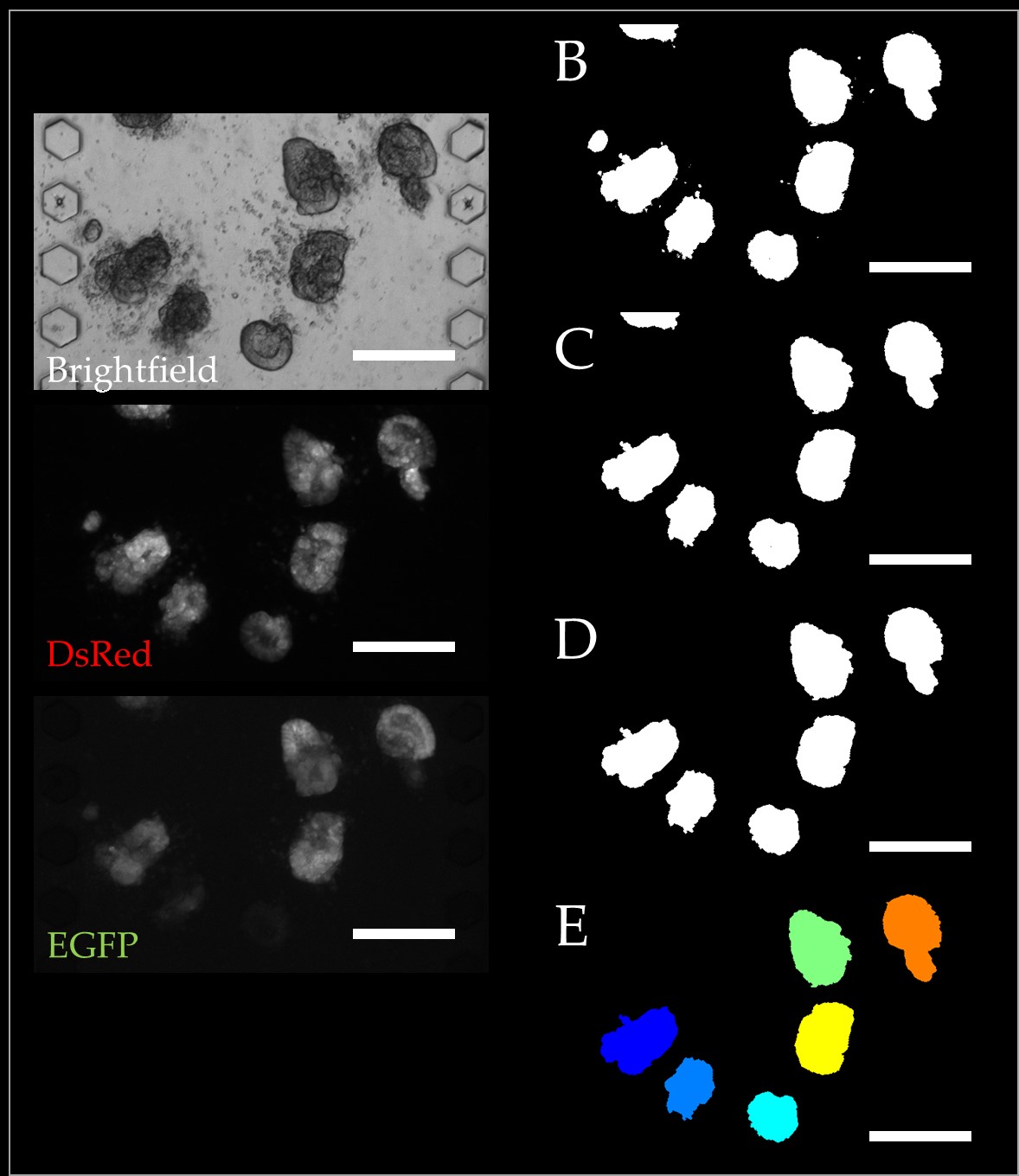

Supplement: S1 Fig — (A) Raw images from brightfield microscopy and fluorescence microscopy of EGFP and DsRed of the sample field of colonoids. (B) A threshold for the processed image was automatically determined by minimum cross entropy thresholding.[38] (C) In the resultant binary image, all objects with a total area less than 1000 μm2 were removed and all interior holes within objects were filled to generate a mask of the segmented colonoids. (D) Large cellular debris was then removed from the images. Cellular debris was defined objects with brightfield segmentation boundaries that were 20% larger than the segmentation boundary obtained from the red fluorescence channel. Colonoids touching the edges of the image were also removed. (E) Finally, each of the colonoids were labelled with a color code for subsequent measurements on that colonoid. Scale bars are 250 μm. (JPG) [file pone.0153795.s001.jpg]

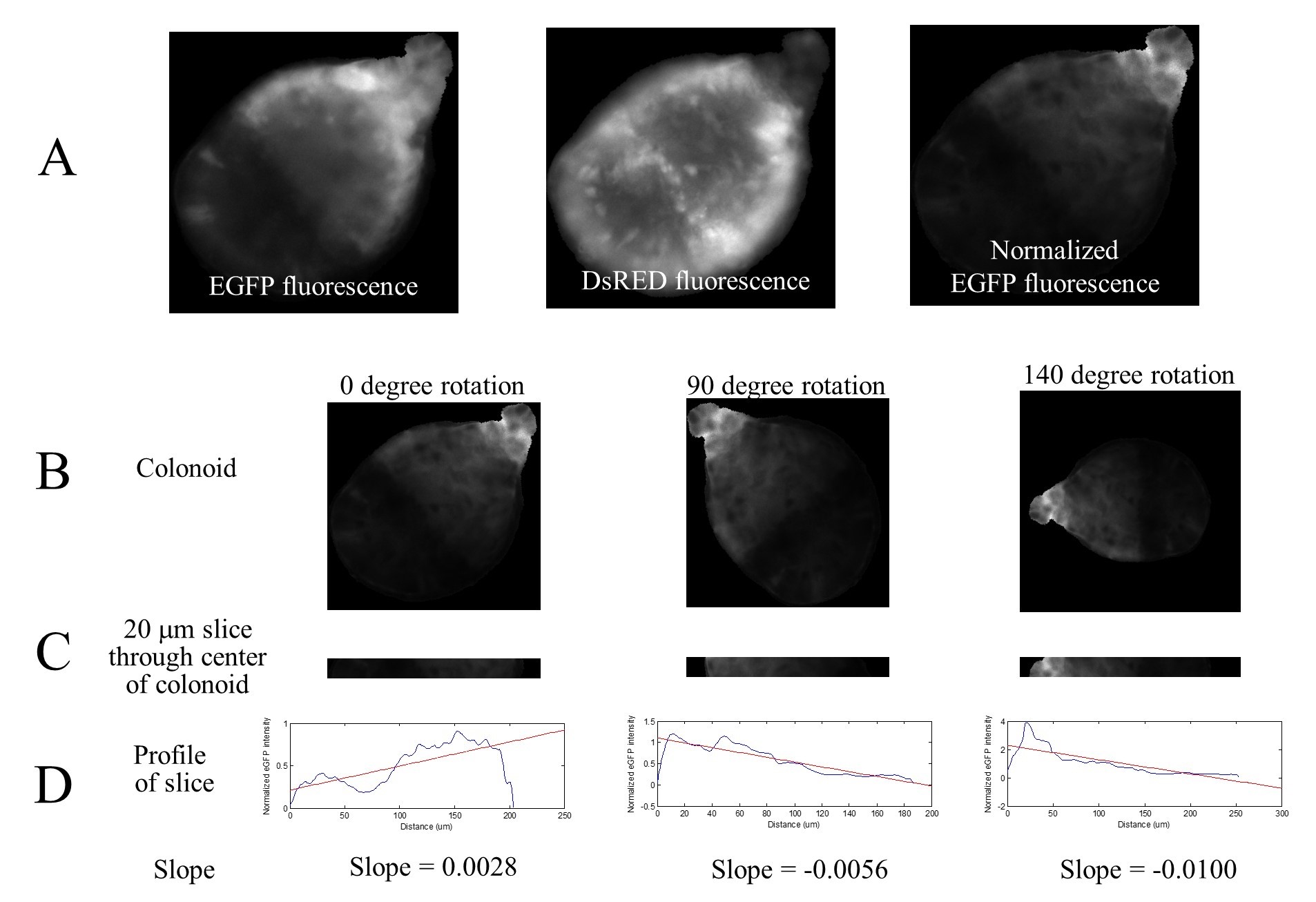

Supplement: S2 Fig — (A) Images of the DsRed fluorescence, EGFP fluorescence, and EGFP divided by DsRed fluorescence. (B) The EGFP divided by DsRed image was rotated every one degree over 180 degrees. (C) A 20-μm horizontal slice through the center of the colonoid was identified. (D) The intensity profile along the 20- μm slice was calculated and a linear fit was performed on the intensity profile to obtain the slope of the best-fit line. (JPG) [file pone.0153795.s002.jpg]

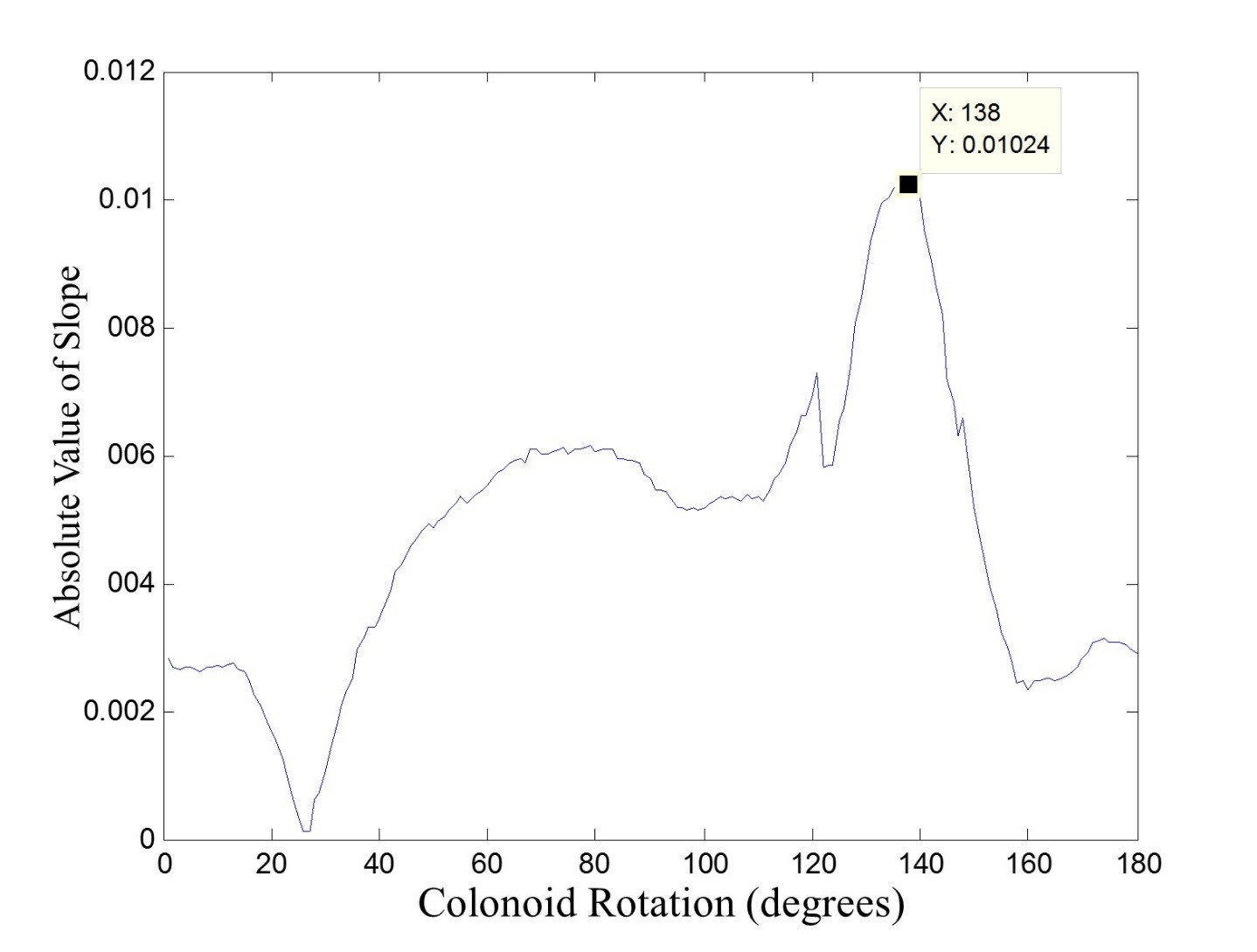

Supplement: S3 Fig — The colonoid rotation angle was plotted against the absolute value of the slope. The rotated image that produced the largest absolute value of the slope was identified. This angle of the rotated image and sign of the slope determined the direction of colonoid polarization. The absolute value of the slope was used as the magnitude of the polarization. In this example the angle of polarization was 138 degrees with a magnitude of 0.01. (JPG) [file pone.0153795.s003.jpg]

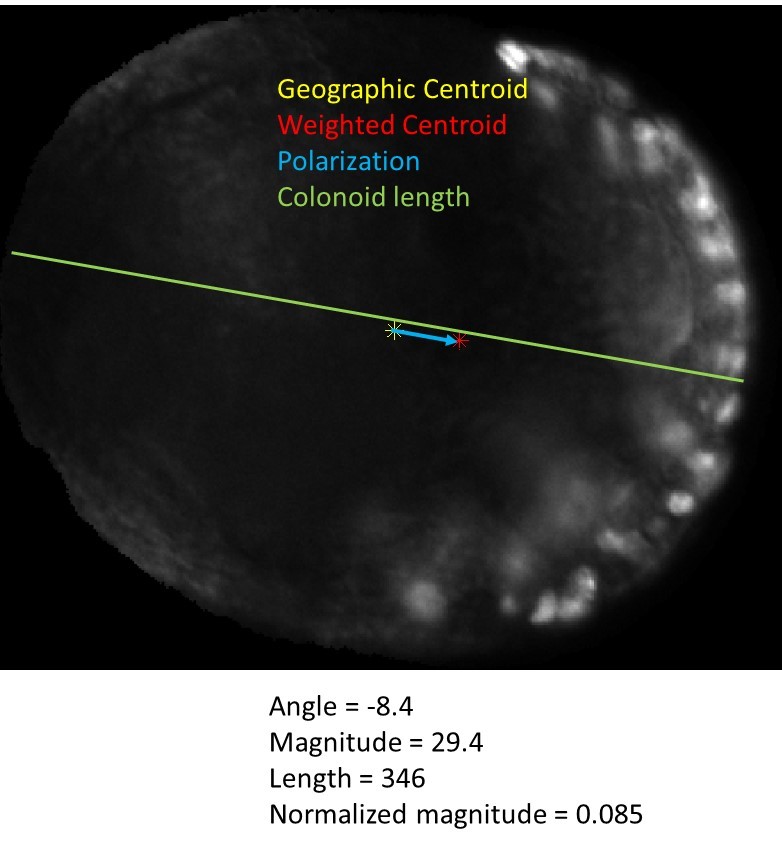

Supplement: S4 Fig — Shown is the EdU fluorescence image of a colonoid. The geographic centroid (yellow asterisk) was obtained from the Hoechst 33342 image (not shown). The EdU-intensity weighted centroid (red asterisk) was also calculated. The angle of polarization was the angle of the vector (blue arrow) that pointed from the geographic centroid to the intensity weighted centroid. The magnitude of the vector was normalized to the colonoid length (346 μm in this example). (JPG) [file pone.0153795.s004.jpg]

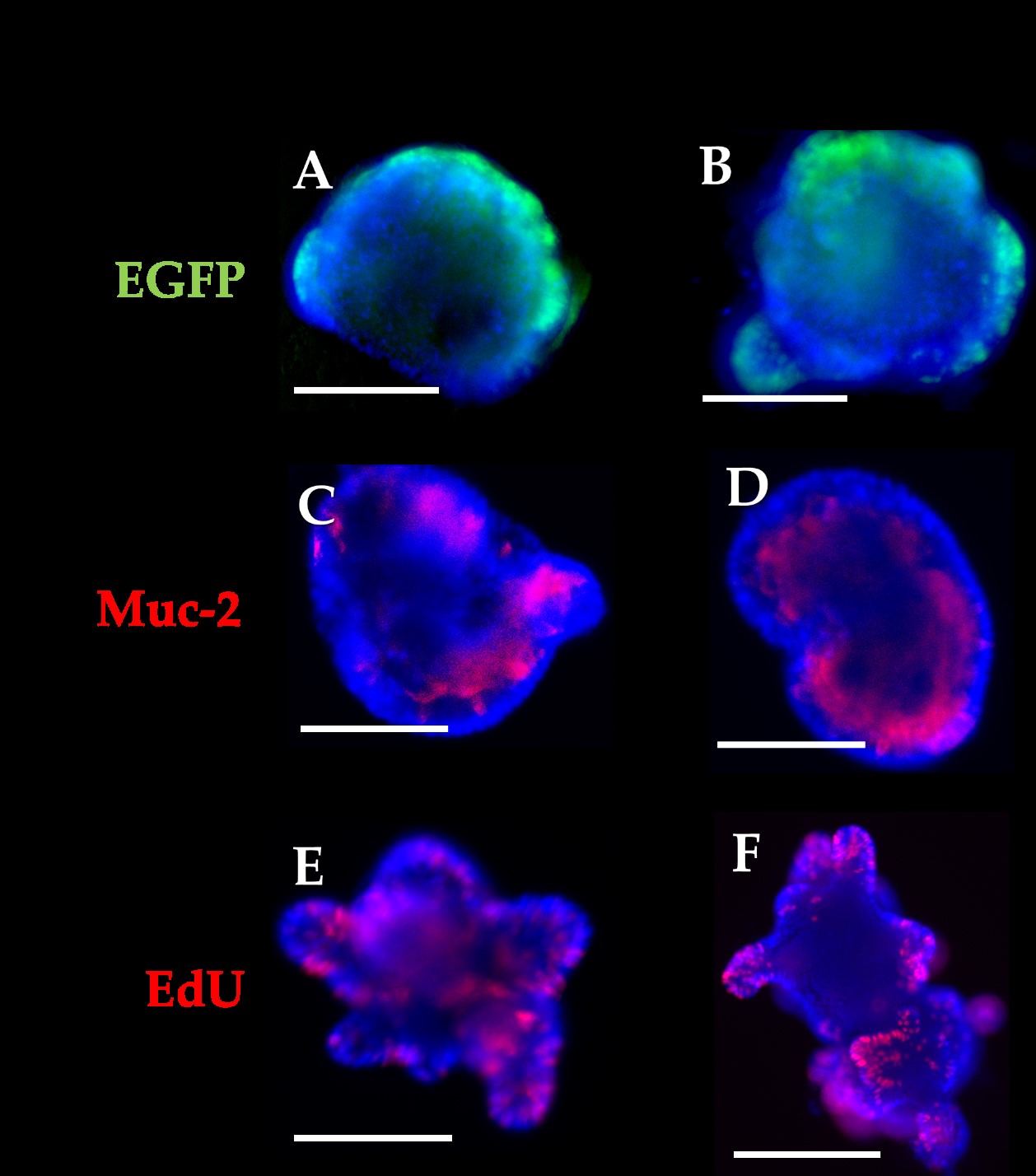

Supplement: S5 Fig — EGFP fluorescence is depicted in green in panels A and B. Muc-2 immunofluorescence is shown in red panels C and D while EdU-based fluorescence is also marked as red panels E and F. Hoechst 33342 fluorescence is blue in all panels. Colonoids were cultured for 5 d in a microchannel (A, C, E) or multiwell plate (B, D, F). The scale bars are 150 μm. (JPG) [file pone.0153795.s005.jpg]

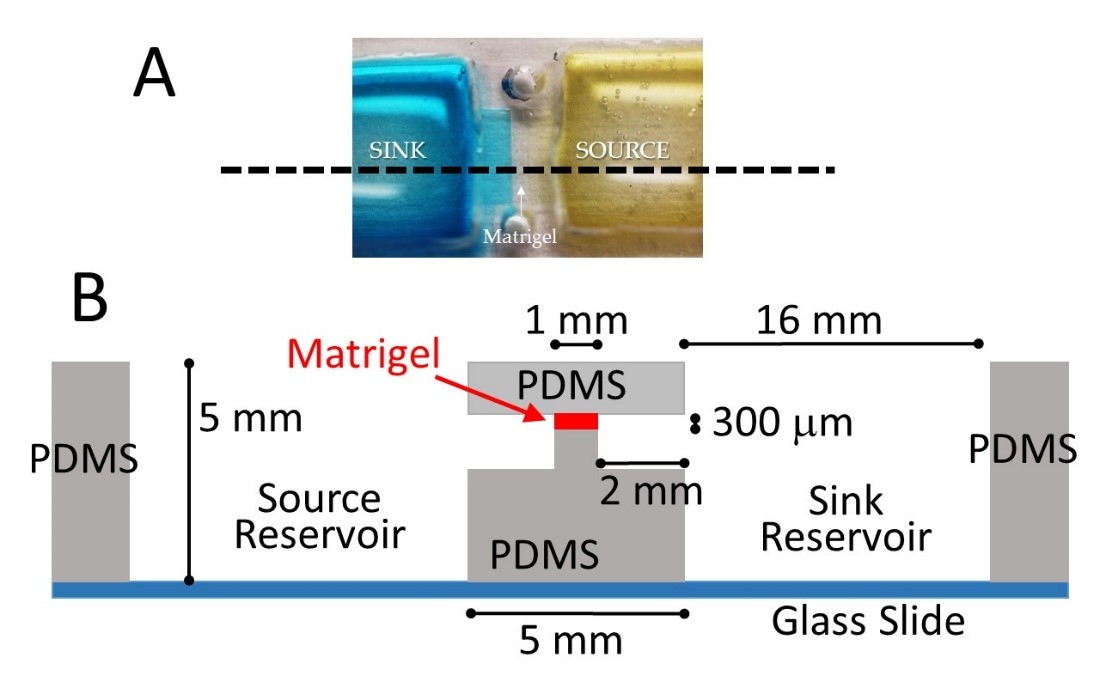

Supplement: S6 Fig — Panel A shows the device of Fig 1A with a dashed black line depicting the location of the cross section shown in panel B. (JPG) [file pone.0153795.s006.jpg]

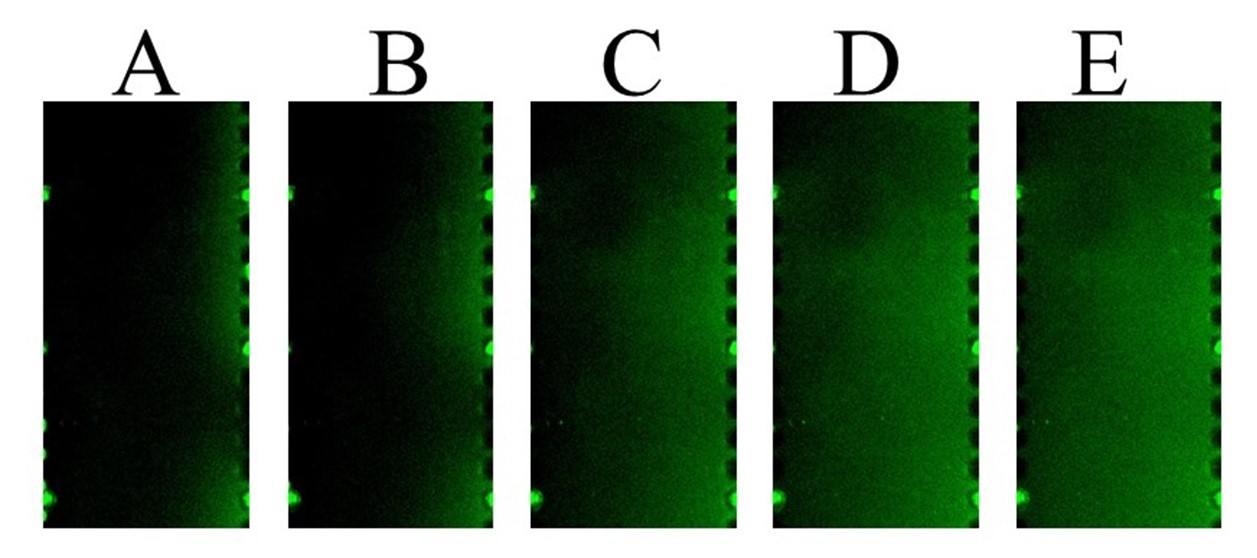

Supplement: S7 Fig — Fluorescein-dextran (40 kD) was loaded into the source only. The fluorescence across the microchannel was then measured. A) Microchannel image immediately after fluorescein dextran placement into the microchannel. B-E) Images of the channel at 1 (B), 6 (C), 12 (D), and 24 (E) h after placing fluorescein dextran into the source. (JPG) [file pone.0153795.s007.jpg]

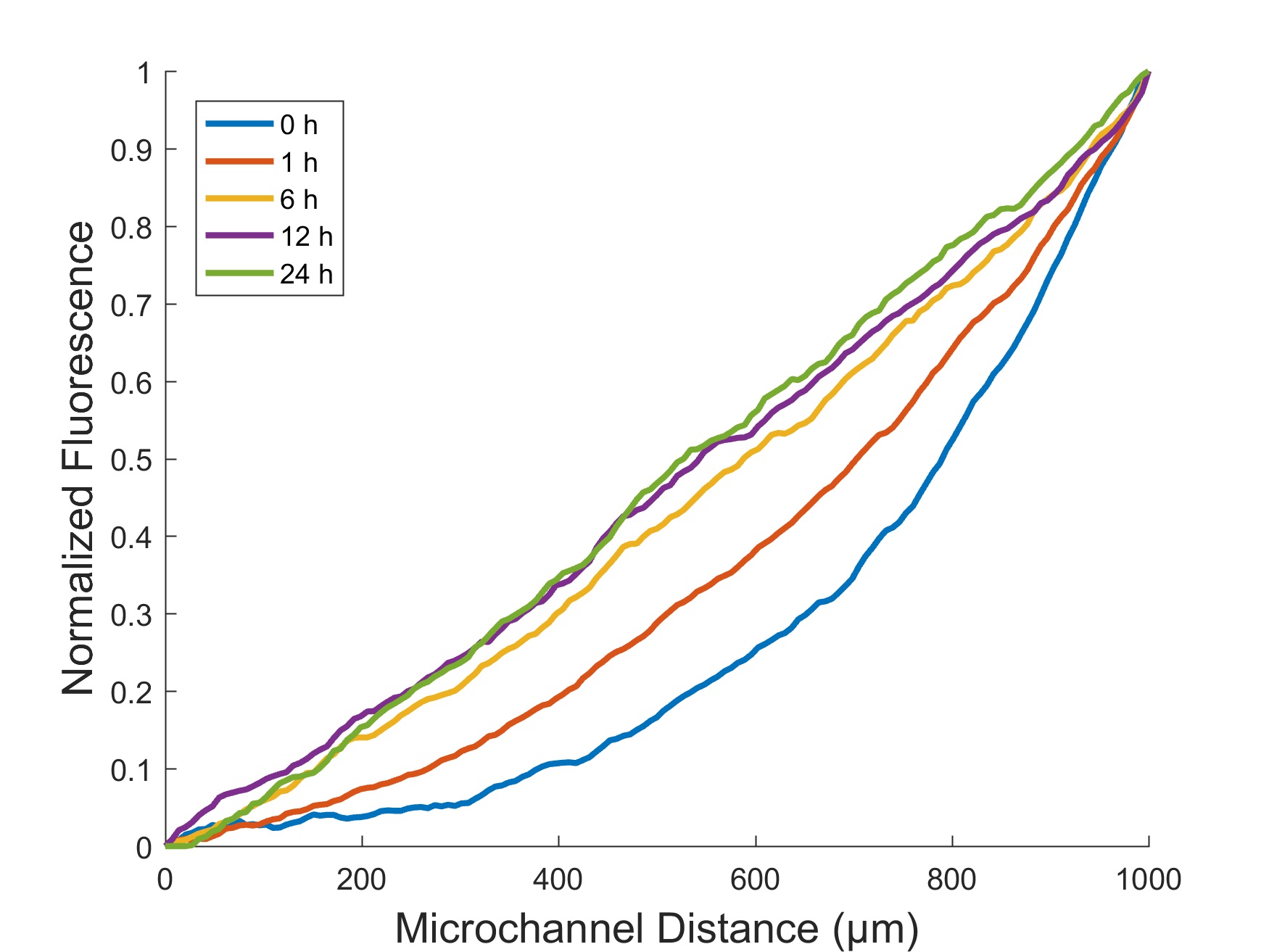

Supplement: S8 Fig — Fluorescein-dextran (40 kD) was loaded into the source. The fluorescence across the microchannel was immediately measured and is labeled as time 0. The fluorescence was again measured at 1, 6, 12, and 24 h. (JPG) [file pone.0153795.s008.jpg]

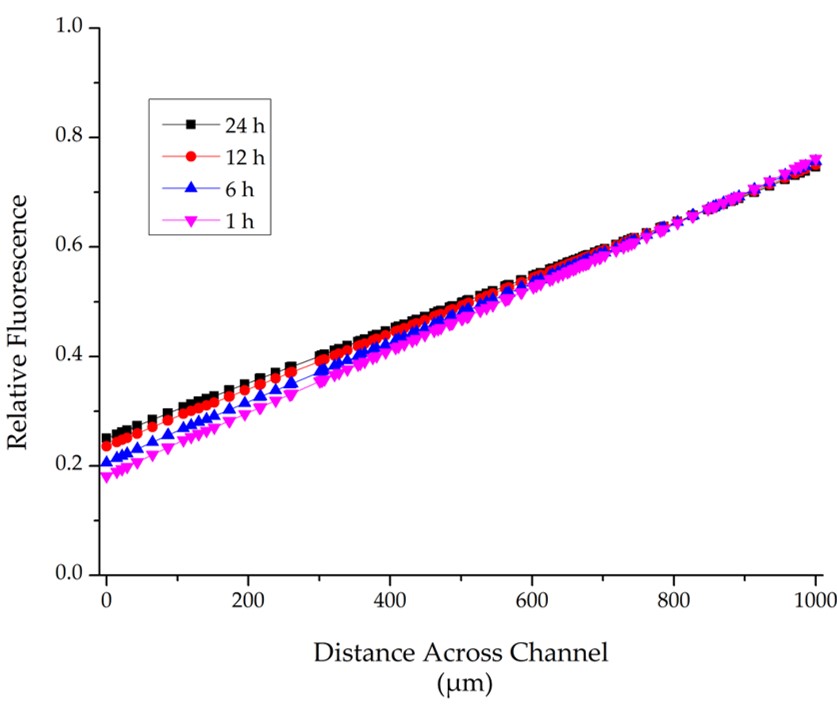

Supplement: S9 Fig — Shown is the fluorescence intensity vs the distance along the microchannel at varying time points after replacement of the source and sink reservoir fluids (marked as time 0 on the graph). The gradient was allowed to form for 24 h and then the reservoir fluids replaced at time 0. The boundary of the sink and Matrigel was designated as 0 μm and that at the Matrigel and source as 1000 μm. (JPG) [file pone.0153795.s009.jpg]

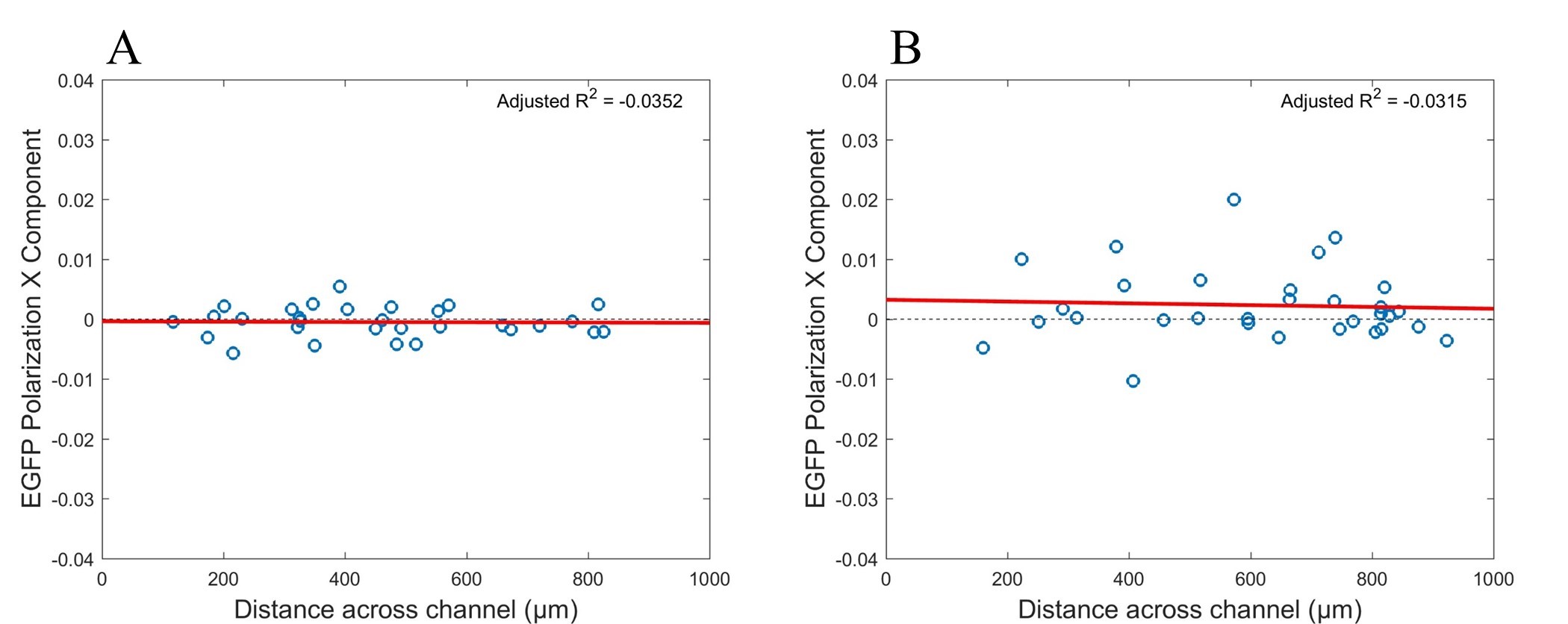

Supplement: S10 Fig — The centroid location of the colonoid fragment in the multiwell plate or microchannel (between the source and sink) was calculated from the DsRed fluorescence. The centroid distance from the Matrigel:sink interface (located at 0 μm) was then plotted against the x-component of the EGFP polarization vector. The Matrigel:source interface was located at 1000 μm. A straight line was fit to the data points (solid line). The images are data from the multiwell plate (A) or microchannel without a gradient (B). (JPG) [file pone.0153795.s010.jpg]

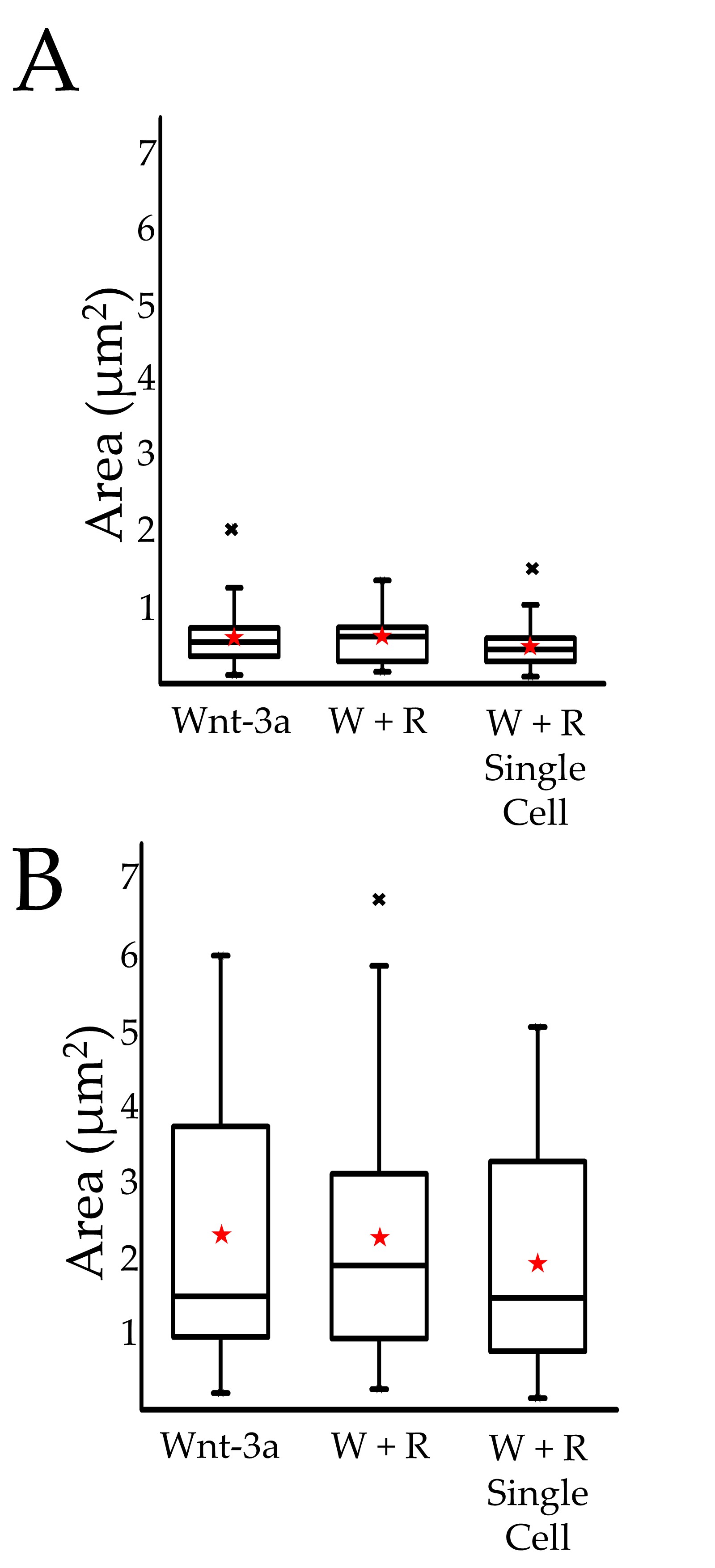

Supplement: S11 Fig — Boxplots were used to represent the DsRed fluorescence area of the colonoids for two gradient conditions (Wnt-3a alone and Wnt-3a/Rspondin1 (W+R)) starting with colonoid fragments and the W+R condition starting with single cells. The non-normal distribution of the colonoid area is represented as μm2 (× 104). For the boxplots, the red star indicates the mean of the data, the horizontal line shows the median, and the upper and lower boxes represent the 75% and 25% of the data, respectively. The whiskers extend to the 5% and 95% with the individual points showing outliers. (A) Day 1 and (B) Day 5. (JPG) [file pone.0153795.s011.jpg]

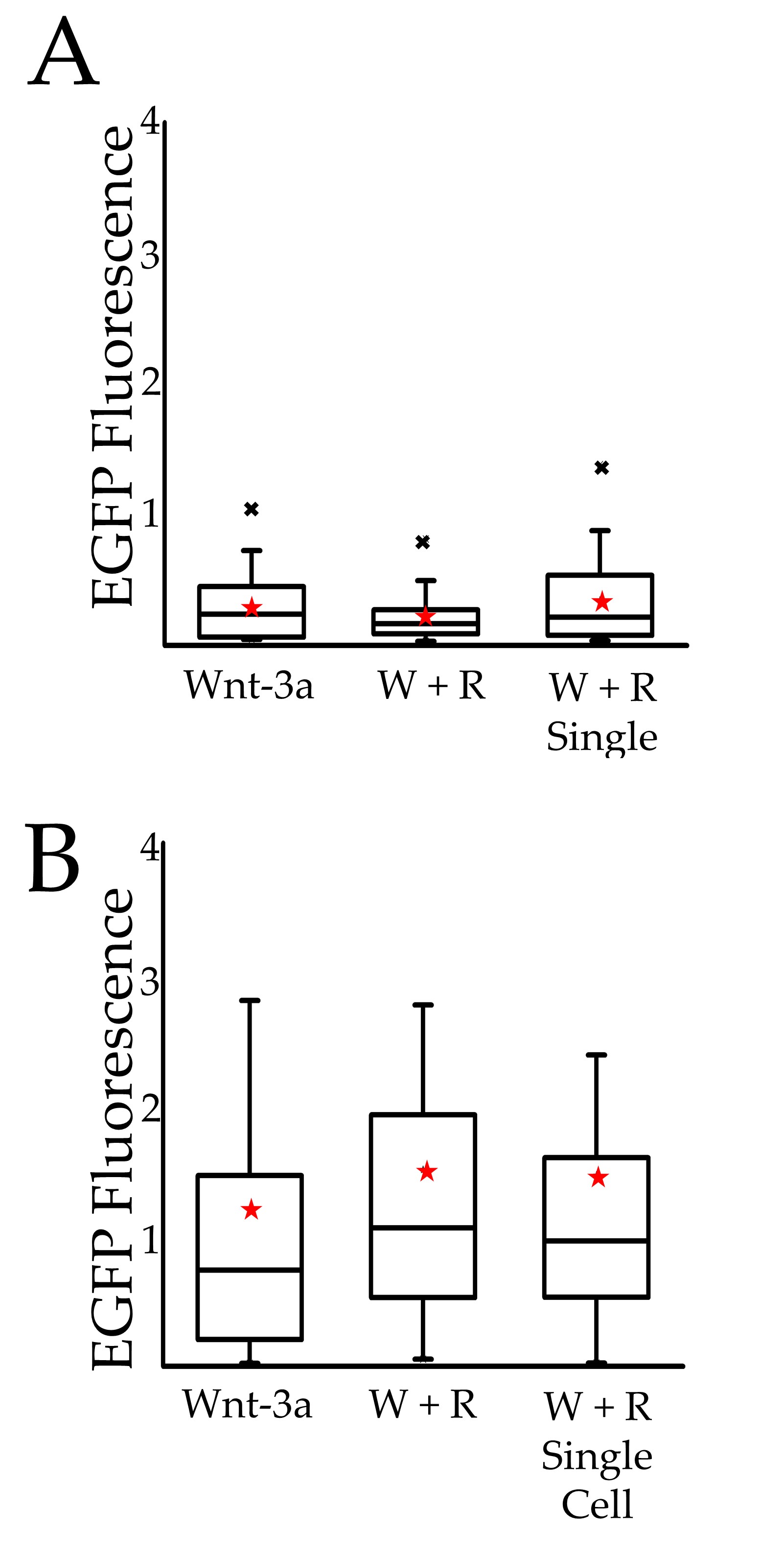

Supplement: S12 Fig — Boxplots were used to represent the integrated EGFP intensity of the colonoids for the two gradient conditions (Wnt-3a alone and Wnt3a/Rspondin1 (W+R)) starting with colonoid fragments and the W+R condition starting with single cells. The non-normal distribution of the colonoid integrated EGFP fluorescence intensity is represented as RFUs (× 105). For the boxplots, the black star indicates the mean of the data, the bar shows the median, and the upper and lower boxes represent the 75% and 25% of the data, respectively. The whiskers extend to the 5% and 95% with the individual points showing outliers. (A) Day 1 and (B) Day 5. (JPG) [file pone.0153795.s012.jpg]

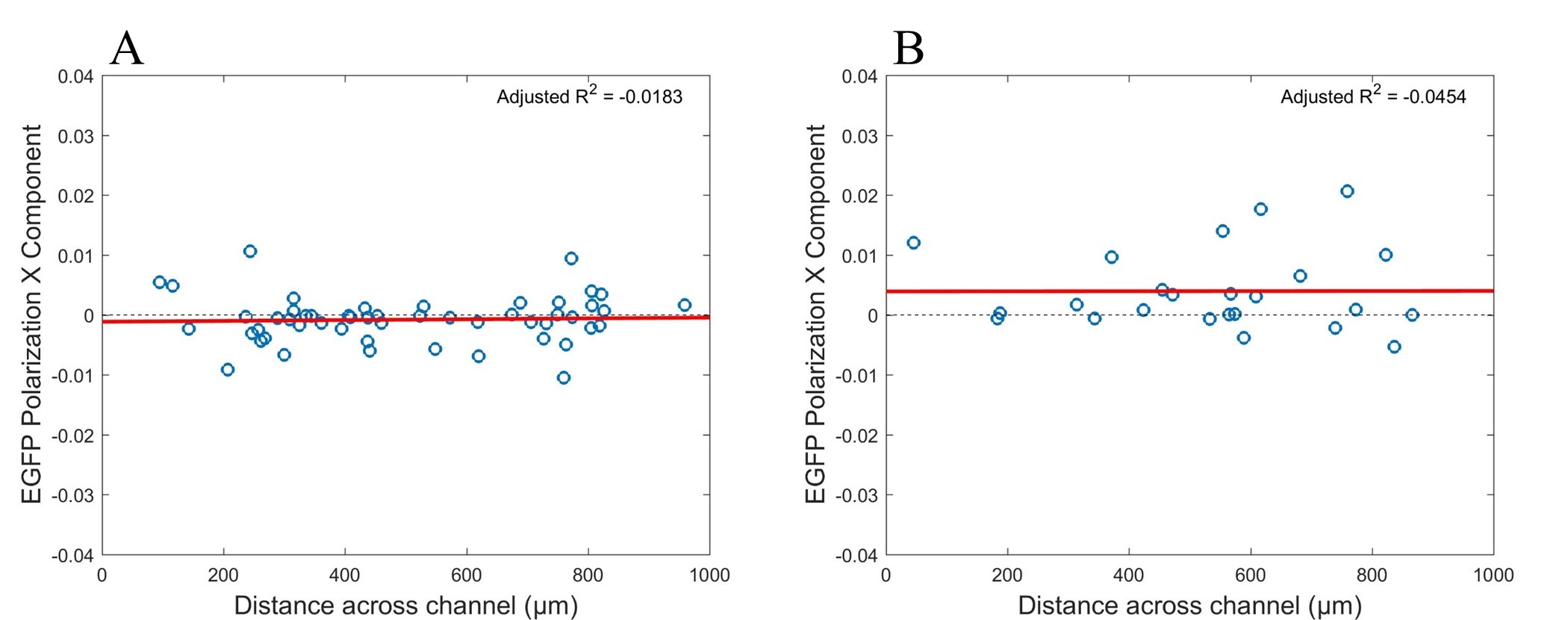

Supplement: S13 Fig — The centroid location of the colonoid in the microchannel (between the source and sink) was calculated from the DsRed fluorescence. The centroid distance from the Matrigel:sink interface (located at 0 μm) was then plotted against the x-component of the EGFP polarization vector. The Matrigel:source interface was located at 1000 μm. A straight line was fit to the data points (solid line). The images are data from the Wnt-3a (A) or Wnt-3a/Rspondin1 (B) gradient. (JPG) [file pone.0153795.s013.jpg]

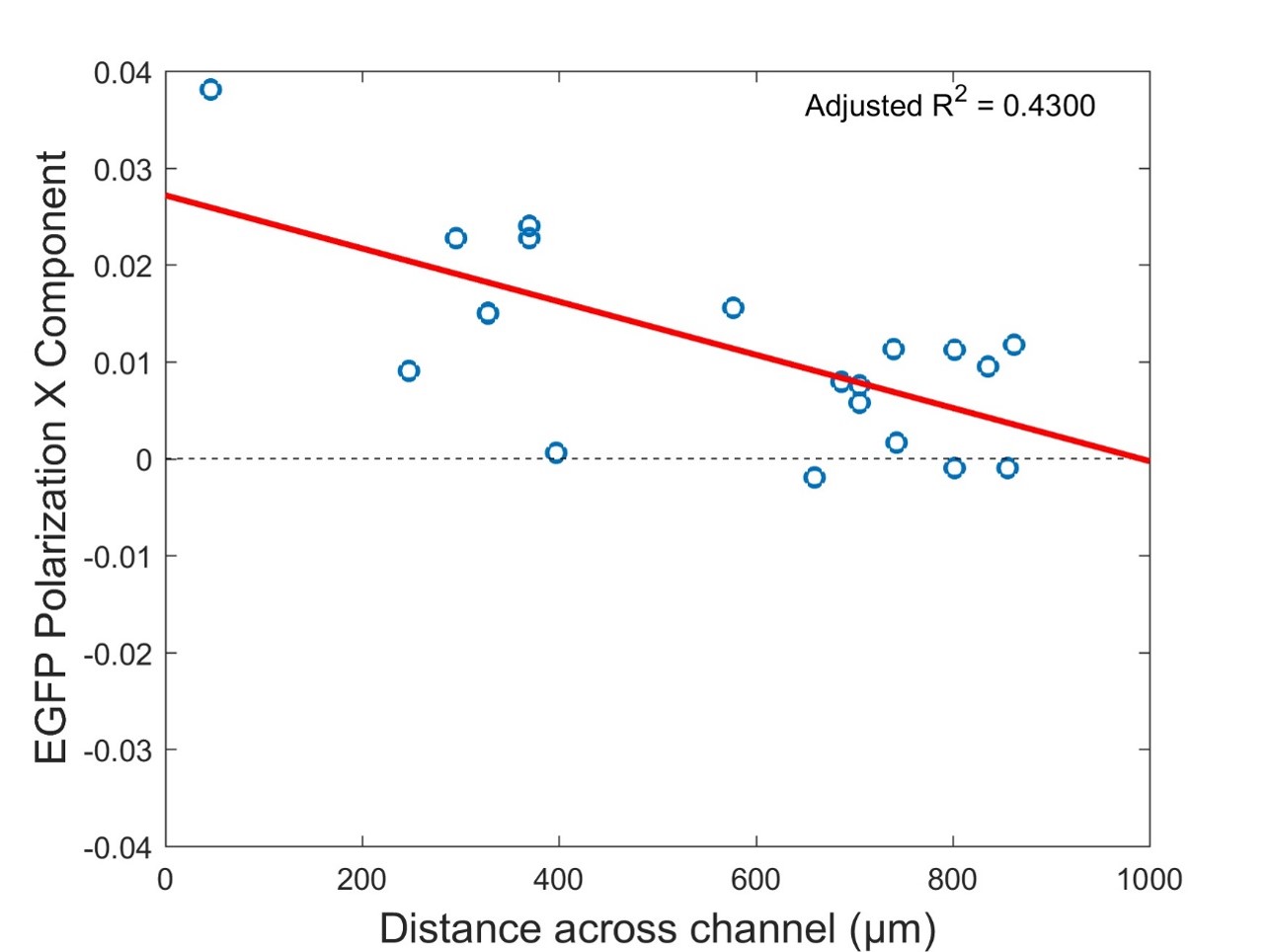

Supplement: S14 Fig — The stem-cell location in the microchannel between the source and sink was plotted against the x-component of the EGFP polarization vector. A straight line was fit to the data points (solid line). (JPG) [file pone.0153795.s014.jpg]
