# Supplementary material for: In Vitro Polarization of Colonoids to Create an Intestinal Stem Cell Compartment
Source: PLoS One. 2016 Apr 21;11(4):e0153795. doi: 10.1371/journal.pone.0153795 (PMC4839657; doi:10.1371/journal.pone.0153795)
Supplement: S1 Methods — (DOCX) [file pone.0153795.s015.docx]

**Fabrication of the 1-mm Gradient Device**

The microfabricated polydimethylsiloxane (PDMS) gradient device possessed two reservoirs (16×16×5 mm each) connected by a central gradient-generating region (5×1×0.3 mm). The device was constructed in three steps. In the first step, a master mold was fabricated using standard photolithography. A layer of 1002F polymer (300 µm) was spin-coated onto a glass slide and baked at 95 °C for 12 hours. The 1002F layer was irradiated with a UV light source (800 mJ) through a mask to crosslink the 1002F. The 1002F layer was then heated at 120 °C for 1 hour to complete polymerization [1]. Unpolymerized material was removed by rinsing with SU-8 developer. The resulting master mold was vapor-coated with octyltrichlorosilane prior to addition of PDMS to improve demolding. A 1 mm layer of PDMS (Dow Corning, Midland, MI) was poured onto the master and cured by baking at 65 °C for 12 hours. After removal of the cured PDMS from the master, the reservoirs were created by cutting square holes in the PDMS. The PDMS device was then attached to a glass slide coated with a 25 µm layer of PDMS after each had been plasma treated for 2 min. The two structures were aligned and brought into conformal contact to form a permanently bonded assembly. The assembled device was then baked at 95 °C overnight to enhance the bond strength.

**Isolation of Single Colonic Stem Cells**

Epithelial cells were dissociated from whole murine colons using previously described techniques with some modifications [2]. Colons were resected, opened longitudinally and rinsed in PBS. Colons were cut into 10 mm segments and transferred to 3 mM EDTA (Sigma), 10 μM Y-27632 (Selleck) and incubated for 45 min at 4^o^C with gentle agitation. Fragments were transferred into fresh PBS with 10 μM Y-27632 and shaken by hand for 2 min to release the epithelial layer from the submucosa. Epithelium was separated from the remnant mucosa, washed twice in PBS and dissociated to single cells in 0.3 U/mL dispase (Life Technologies), 10 μM Y-27632 in Hank’s Buffered Saline Solution (HBSS, Life Technologies) incubated at 37 °C for 10-14 min while shaking every 2 min. Cell solutions were passed sequentially through 100 μm, 70 μm, and 40 μm pore-size nylon filters before being transferred to intestinal stem cell (ISC) culture media (Advanced DMEM/F12 (Life Technologies), N2 (1×, Life Technologies), B27 (1×, Life Technologies), Glutamax (1×, Life Technologies), penicillin (100 unit/mL, Life Technologies), and streptomycin (100 µg/mL, Life Technologies), 10 mM HEPES (Life Technologies), 10 μM Y27632 (Selleck Chemicals) and 500 mM N -acetyl- cysteine (Sigma)). Isolated cells were stained with Sytox Blue (Life Technologies) and Annexin V Pacific Blue (Biolegend) to label dead cells. Fluorescence-activated cell sorting (FACS) was performed using a SH800 FACS Instrument (Sony) to isolate Sox9^EGFP^low:CAG^DsRed^ stem cells using previously described techniques. Cells were sorted into ISC culture media prior to use in gradient devices. The accuracy of the sort was assessed by post-sort analysis of 1000 sorted Sox9^EGFP^low:CAG^DsRed^ stem cells, resulting in an overall purity of 3%.

**COMSOL Modeling**

COMSOL Multiphysics (Version 4.4, COMSOL Inc., Burlington, MA) was used to model the molecular gradient across the microdevice using the experimentally determined diffusion coefficient of 7.2 × 10^−11^ m^2^ s^−1^. The microdevice was modeled in the COMSOL environment to have identical dimensions as the experimental microdevice. The “Transport of Diluted species” module was used with the assumption that the diffusion was isotropic, there was no flux at the device boundaries and that there was no convective mixing. The initial source concentration was set to 120 ng/mL, corresponding to the Wnt-3a concentration used experimentally, and the source concentration was set to 0 ng/mL. The source and sink concentrations were re-initialized at 24 h time points to mimic the experimental replenishment of source and sink on the microdevice. The model was computed at 1 h time points for 96 hours.

**References**

1. J. H. Pai, Y. Wang, G. T. Salazar, C. E. Sims, M. Bachman, G. P. Li and N. L. Allbritton, *Analytical Chemistry*, 2007, **79**, 8774-8780.

2. A. D. Gracz, B. J. Puthoff and S. T. Magness, *Methods in molecular biology (Clifton, N.J.)*, 2012, **879**, 89-107.

3. A. A. Ahmad, Y. Wang, C. E. Sims, S. T. Magness and N. L. Allbritton, *RSC Advances*, 2015, **5**, 74881-74891.
