# Supplementary material for: In Vitro Polarization of Colonoids to Create an Intestinal Stem Cell Compartment
Source: PLoS One. 2016 Apr 21;11(4):e0153795. doi: 10.1371/journal.pone.0153795 (PMC4839657; doi:10.1371/journal.pone.0153795)
Supplement: S1 Table — Six devices were used for each microchannel experiment and three wells for each multiwell-plate experiment. (DOCX) [file pone.0153795.s016.docx]

**Table S1.** Area occupied by each colonoid in a 2-D image slice in the absence of a gradient after 1 and 5 days of culture in the microchannel or multiwell plate. Six devices were used for each microchannel experiment and three wells for each multiwell-plate experiment.

| Conditions | Day | Number of Crypts/Colonoids | Quartile 1 (µm^2^) | Median (µm^2^) | Quartile 3 (µm^2^) | |
| --- | --- | --- | --- | --- | --- | --- |
| Microchannel | 1 | 25 | 2,950 | 4,225 | 6,040 | |
| Multiwell Plate | 1 | 29 | 3,185 | 4,660 | 6,445 | |
| Microchannel | 5 | 25 | 6,352 | 13,236 | 29,520 |  |
| Multiwell Plate | 5 | 29 | 7,538 | 12,752 | 36,637 |  |
