# Supplementary material for: In Vitro Polarization of Colonoids to Create an Intestinal Stem Cell Compartment
Source: PLoS One. 2016 Apr 21;11(4):e0153795. doi: 10.1371/journal.pone.0153795 (PMC4839657; doi:10.1371/journal.pone.0153795)
Supplement: S2 Table — For the microchannel, n = 20 colonoids in 5 microchannels and for the multiwell plate, n = 20 colonoids in 3 wells. (DOCX) [file pone.0153795.s017.docx]

**Table S2.** Percentage of each colonoid with Muc-2 immunofluorescence in a 2-D image slice in the absence of a gradient after 5 days of culture on the microchannel and multiwell plate. For the microchannel, n = 20 colonoids in 5 microchannels and for the multiwell plate, n = 20 colonoids in 3 wells.

| Conditions | Day | Number of Colonoids | Average % of pixels with Muc-2 immunofluorescence | % of colonoids with >10% of the pixels positive for Muc-2 immunofluorescence |
| --- | --- | --- | --- | --- |
| Microchannel | 5 | 20 | 32 ± 7% | 90 ± 5% |
| Multiwell Plate | 5 | 20 | 28 ± 9% | 92 ± 7% |
