# Supplementary material for: In Vitro Polarization of Colonoids to Create an Intestinal Stem Cell Compartment
Source: PLoS One. 2016 Apr 21;11(4):e0153795. doi: 10.1371/journal.pone.0153795 (PMC4839657; doi:10.1371/journal.pone.0153795)
Supplement: S3 Table — (DOCX) [file pone.0153795.s018.docx]

**Table S3.** Percentage of each colonoid with EGFP fluorescence in a 2-D image slice in the absence of a gradient after 5 days of culture on the microchannel and multiwell plate.

| Conditions | Day | Number of Colonoids | Average % of pixels with EGFP fluorescence | % of colonoids with >25% of the pixels positive for EGFP fluorescence |
| --- | --- | --- | --- | --- |
| Microchannel | 5 | 15 | 66 ± 17% | 83 ± 8% |
| Multiwell Plate | 5 | 15 | 69 ± 14% | 85 ± 9% |
