# Supplementary material for: In Vitro Polarization of Colonoids to Create an Intestinal Stem Cell Compartment
Source: PLoS One. 2016 Apr 21;11(4):e0153795. doi: 10.1371/journal.pone.0153795 (PMC4839657; doi:10.1371/journal.pone.0153795)
Supplement: S4 Table — (DOCX) [file pone.0153795.s019.docx]

**Table S4**. EGFP fluorescence intensity of colonoids in a 2-D image slice in the absence of a gradient after 1 and 5 days of culture in the microchannel or multiwell plate.

| Conditions | Day | Number of Crypts/Colonoids | Quartile 1 | Median | Quartile 3 |
| --- | --- | --- | --- | --- | --- |
| Microchannel | 1 | 25 | 10,255 | 22,100 | 59,860 |
| Multiwell Plate | 1 | 25 | 9,970 | 25,800 | 57,580 |
| Microchannel | 5 | 25 | 46,611 | 118,822 | 199,382 |
| Multiwell Plate | 5 | 25 | 64,285 | 133,490 | 187,588 |
