# Supplementary material for: In Vitro Polarization of Colonoids to Create an Intestinal Stem Cell Compartment
Source: PLoS One. 2016 Apr 21;11(4):e0153795. doi: 10.1371/journal.pone.0153795 (PMC4839657; doi:10.1371/journal.pone.0153795)
Supplement: S5 Table — (DOCX) [file pone.0153795.s020.docx]

**Table S5.** Percentage of each colonoid with EdU fluorescence in a 2-D image slice in the absence of a gradient after 5 days of culture on the microchannel and multiwell plate.

| Conditions | Day | Number of Colonoids | Average % of pixels with EdU fluorescence | % of colonoids with >25% of the pixels positive for EdU fluorescence | |
| --- | --- | --- | --- | --- | --- |
| Microchannel | 5 | 15 | 57 ± 10% | 96 ± 3% |  |
| Multiwell Plate | 5 | 15 | 64 ± 14% | 92 ± 7% |  |
