# Supplementary material for: In Vitro Polarization of Colonoids to Create an Intestinal Stem Cell Compartment
Source: PLoS One. 2016 Apr 21;11(4):e0153795. doi: 10.1371/journal.pone.0153795 (PMC4839657; doi:10.1371/journal.pone.0153795)
Supplement: S6 Table — (DOCX) [file pone.0153795.s021.docx]

**Table S6.** The correlation coefficient, *p*-value, slope and adjusted R^2^ value for plots of the colonoid microchannel location vs the x component of the EGFP polarization vector.

|  | **Correlation Coefficient** | ***p*-Value** | **Slope (µm^-1^)** | **Adjusted R^2^ Value** |
| --- | --- | --- | --- | --- |
| **Colonoids in Multiwell Plate** | -0.0950 | 0.62 | -0.25 ± 4.62 ×10^-6^ | -0.0352 |
| **Colonoids in Microchannel, No Gradient** | 0.1412 | 0.33 | 0.69 ± 4.79 ×10^-6^ | -0.0183 |
| **Colonoids in Microchannel, Wnt-3a Gradient** | -0.1254 | 0.50 | -0.15 ± 1.06 ×10^-5^ | -0.0315 |
| **Colonoids in Microchannel, Wnt-3a/Rspondin1 Gradient** | -0.0409 | 0.85 | 0.01 ± 1.34 ×10^-5^ | -0.0454 |
| **Single Cells in Microchannel, Wnt-3a/Rspondin1 Gradient** | -0.5089 | 0.013 | -2.74 ± 1.36 ×10^-5^ | 0.4300 |

The colonoid centroid location within the microchannel (distance from the sink) was plotted against the x component of the polarization vector based on EGFP fluorescence (see Figures S10, S13, and S14). The Spearman’s rank correlation coefficient was calculated and a permutation test was performed (*p*-value) for all conditions.^1^ The *p*-value tests the null hypothesis that “there is no positive or negative correlation between the colonoid location in the microchannel and the x-component of the EGFP polarization vector.” Additionally, the slope and adjusted coefficient of determination (R^2^) was computed from a linear regression performed on the data sets.^2^ The listed error values for the slope represent the 95% confidence level. There was a statistically significant negative correlation between colonoid position in the channel and x polarization value among colonoids originating from single cells in a Wnt-3a/Rspondin1 gradient. All other conditions exhibited no significant correlation between colonoid position and EGFP polarization.

**References**

1. E. C. Fieller, H. O. Hartley and E. S. Pearson, *Biometrika*, 1957, **44**, 470-481.

2. N. R. Draper and H. Smith, *Applied regression analysis*, John Wiley & Sons, 2014.
