# Supplementary material for: In Vitro Polarization of Colonoids to Create an Intestinal Stem Cell Compartment
Source: PLoS One. 2016 Apr 21;11(4):e0153795. doi: 10.1371/journal.pone.0153795 (PMC4839657; doi:10.1371/journal.pone.0153795)
Supplement: S7 Table — (DOCX) [file pone.0153795.s022.docx]

**Table S7.** Integrated EGFP intensity of a 2-D image slice of colonoids developed within a Wnt-3a gradient after 1 and 5 days of culture on the microdevice.

| Conditions | Day | Number of Colonoids | Quartile 1 | Median | Quartile 3 |
| --- | --- | --- | --- | --- | --- |
| Wnt-3a | 1 | 35 | 6,769 | 23,916 | 43,490 |
| Wnt-3a | 5 | 28 | 25,445 | 73,591 | 143,216 |
