# Supplementary material for: In Vitro Polarization of Colonoids to Create an Intestinal Stem Cell Compartment
Source: PLoS One. 2016 Apr 21;11(4):e0153795. doi: 10.1371/journal.pone.0153795 (PMC4839657; doi:10.1371/journal.pone.0153795)
Supplement: S8 Table — (DOCX) [file pone.0153795.s023.docx]

**Table S8.** Integrated EGFP intensity of a 2-D image slice of colonoids developed within a Wnt-3a + Rspondin1 gradient after 1 and 5 days of culture on the microdevice.

| Conditions | Day | Number of Colonoids | Quartile 1 | Median | Quartile 3 |
| --- | --- | --- | --- | --- | --- |
| W + R | 1 | 40 | \|  \| \| --- \| \| 10,074 \| \|  \| | 16,576 | 27,275 |
| W + R | 5 | 35 | \|  \| \| --- \| \| 53,542 \| \|  \| | 105,823 | 189,950 |
