# Supplementary material for: In Vitro Polarization of Colonoids to Create an Intestinal Stem Cell Compartment
Source: PLoS One. 2016 Apr 21;11(4):e0153795. doi: 10.1371/journal.pone.0153795 (PMC4839657; doi:10.1371/journal.pone.0153795)
Supplement: S9 Table — (DOCX) [file pone.0153795.s024.docx]

**Table S9.** Integrated EGFP intensity of a 2-D image slice of colonoids developed from single cells within a Wnt-3a + Rspondin1 gradient after 1 and 5 days of culture on the microdevice.

| Conditions | Day | Number of Cells/Colonoids | Quartile 1 | Median | Quartile 3 |
| --- | --- | --- | --- | --- | --- |
| W + R | 1 | 37 | 7,751 | 20,816 | 44,503 |
| W + R | 5 | 30 | 52,901 | 95,734 | 159,551 |
